# Detection of autoimmune antibodies in localized scleroderma by synthetic oligonucleotide antigens

**DOI:** 10.1371/journal.pone.0195381

**Published:** 2018-04-11

**Authors:** Simone Samuelsen, Christian Damsgaard Jørgensen, Elizabeth D. Mellins, Kathryn S. Torok, Kira Astakhova

**Affiliations:** 1 Department of Chemistry, Technical University of Denmark, Kongens Lyngby, Region Hovedstaden, Denmark; 2 Department of Mathematics and Computer Science, University of Southern Denmark, Odense, Region Syddanmark, Denmark; 3 Department of Pediatrics, Program in Immunology, Stanford University School of Medicine, Stanford, California, United States of America; 4 Department of Pediatrics, Division of Rheumatology, Children’s Hospital of Pittsburgh, University of Pittsburgh, Pittsburgh, Pennsylvania, United States of America; Instituto Nacional de Ciencias Medicas y Nutricion Salvador Zubiran, MEXICO

## Abstract

In this study, we developed a series of synthetic oligonucleotides that allowed us to investigate the details on the antigen recognition by autoimmune antibodies in localized scleroderma subjects. Besides dramatically improved analytical specificity of the assay, our data suggests a potential linking for antibodies to DNA to the biological status of disease state in localized scleroderma. Moreover, introducing chemical modifications into short synthetic deoxyribonucleic acid (DNA) and ribonucleic acid (RNA) molecules completely changed the binding titers of corresponding antibodies and their clinical relevance. The strongest observed effect was registered for the localized scleroderma skin damage index (LoSDI) on the IgG antibodies to TC dinucleotide-rich double-stranded antigen (p < 0.001). In addition to providing valuable tools for diagnosis of clinically relevant biomarkers, we believe that this work opens up new opportunities for research on antibodies to nucleic acids in localized scleroderma and other autoimmune diseases.

## Introduction

Localized Scleroderma (LS) is an autoimmune disease characterized by hardening of the skin with subsequent atrophy, and typically in childhood onset this occurs in linear bands along the lines of Blaschko. It frequently affects the underlying subcutis, fascia, bones and muscles, resulting in growth defects, especially in childhood onset disease [1, 2]. The diagnosis of LS, also termed morphea, is typically delayed due to its under-recognition and misdiagnosis, with an average delay of disease onset to diagnosis of 12 months [2, 3]. Diagnosis is usually made by a physician more familiar with the condition, a dermatologist or rheumatologist, and may be augmented by the performance of a skin biopsy. The histological examination of the skin demonstrates edematous changes in collagen and the presence of a lymphoplasmacytic infiltrate in the dermis, perivascular and peribulbar/eccrine glands during the early phase, and hyalinization of collagen with loss of skin appendages in later stages of LS [1, 3].

Although the pathogenesis of LS remains unclear, there is evidence supporting it as an autoimmune disease, including a positive auto-antibody profile, concurrent associated autoimmune diseases (vitiligo and arthritis), and family history of autoimmune disease [4]. Autoantibodies, which are commonly identified in LS patients include antinuclear antibodies (ANA), anti-single-stranded deoxyribonucleic acid antibody (anti-ssDNA Ab), and anti-histone antibody (AHA) [5].

There are no diagnostic laboratory tests for LS but there is a high prevalence of autoantibodies including ANA, which could be supportive of the diagnosis [5]. According to the literature, a positive ANA is found in 42 to 73% of LS subjects and has been associated with an increased risk for disease complications [6, 7]. The prevalence of ss-DNA and AHA in LS is approximately 50% [8] and both are associated with disease severity features, such as deep muscle involvement, joint contractures and increase number of lesions [7, 9].

**Fig 1:**
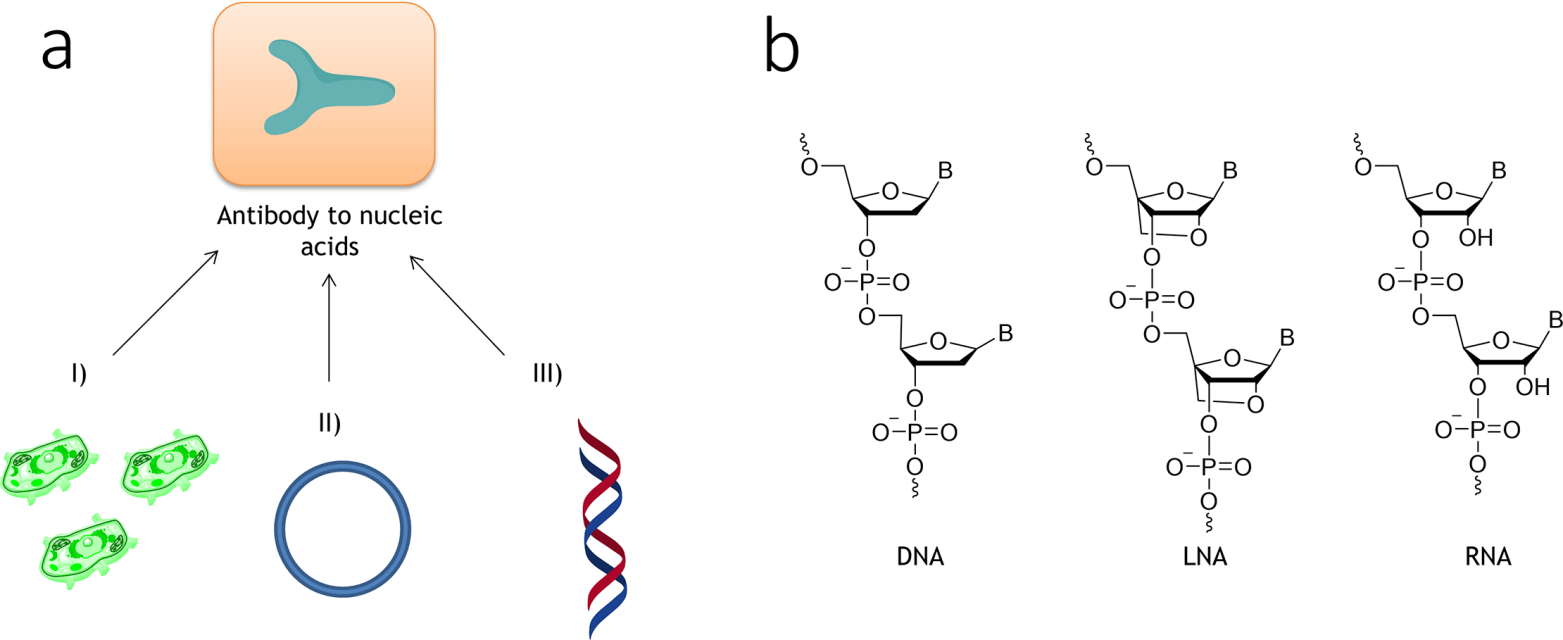
Current methods for the detection of a-DNA and chemical structures of nucleic acid antigens. (a) Current detection methods for anti-DNA: *HEp-2 cell* assay (I), ELISA using plasmid DNA antigen (II), and the novel assay suggested in this work (using synthetic DNA/LNA/RNA; III). (b) Chemical structures of DNA, LNA and RNA nucleotides.

Today, patients suspected with LS are offered ANA, anti-single-stranded deoxyribonucleic acid (anti-ssDNA) and anti-histone tests. For more than 50 years, the immunofluorescent ANA test has been the “gold standard” in the identification of autoimmune disease [6, 10]. When positive in LS patients, ANA has been associated with early disease, increased risk of extra cutaneous manifestations [7, 11] and predictor of flare in LS [12]. However, “the gold standard” lacks specificity as it provides a high frequency of serological positivity related to many other conditions [10, 13]. In LS, anti-ssDNA and anti-histone antibodies are correlated with each other and are associated with more severe disease, as defined by more extensive skin involvement and joint contractors [13–16]. However, Arkachaisiri et al. specifically studied ANA determined by HEp-2 cellular assay in LS and did not find any significant correlations with clinical features in LS (Fig 1A and 1I) [7].

In terms of anti-ssDNA antibodies, a growing number of reports doubt their specificity to autoimmune diseases [17]. This is mainly due to the fact that anti-ssDNA antibodies actively cross-react with other antigens, including phospholipids and the plasma proteins binding them [18]. Anti-double-stranded DNA (anti-dsDNA) antibodies belong to same group of ANA as anti-ssDNA, are common biomarkers in systemic lupus erythematosus (SLE), and show advantageous specificity compared to anti-ssDNA (Fig 1A, III) [14,18, 19].

Once the diagnosis of LS is given, the patient is monitored regularly, and the disease activity is assessed using an indexing system. LS comprises an inflammatory “active” phase and a later, more fibrotic, “damage” phase. The more active phase is associated with elevations in the modified localized scleroderma skin severity index (LoSSI) and physicians’ global assessment of disease activity (PGA-A), whereas the damage phase is associated with an elevation in the localized scleroderma skin damage index (LoSSI) and physicians’ global assessment of disease damage (PGA-D) [20, 21]. These indices are mainly based on the clinical manifestations of LS, and their use requires considerable time, training and operator experience. Having reliable biomarkers that could aid in the diagnosis, disease activity monitoring and outcome prediction of LS would assist in current patient management and therapeutic improvements [22].

In general, specific antibody recognition can be achieved by ssDNA or ribonucleic acid (RNA) molecules called aptamers [22]. Although the use of aptamers as diagnostic tools and therapeutic agents has multiple benefits, only few aptamers are currently undergoing clinical development. Post-SELEX locked nucleic acid (LNA) modification of aptamers is one of the more promising methods to obtain short aptamers with increased biostability, structural diversity, and bioactivity (Fig 1B) [23]. In case of antibody targeting, the sensitivity to chemical composition of aptamers is well known [24]. Nevertheless, the translational role of synthetic DNA and RNA in LS has been limited so far by the lack of confirmed correlations with clinical and biological role of the corresponding antibodies [24, 25].

As an emerging alternative to the rather labor extensive and expensive SELEX, computational techniques are attracting growing attention in the community [25]. In our recent work, we used computational design for the development of novel DNA and LNA/DNA antigens [25]. Next, we proved the efficacy of these short, LNA-labelled DNA in the detection of autoantibodies associated with pediatric SLE [25] a systemic autoimmune disease with multiple manifestations that affects multiple organ systems and is notoriously associated with ANA positivity, as well as ds-DNA positivity. After determining their utility in pediatric SLE, we decided to further expand the spectrum by applying synthetic DNA, LNA, RNA, and their mixmers to the detection of antibodies associated with LS. In this paper we describe the synthesis and the diverse tests of these new antigens, and report the results of statistical analyses of the effects of the clinical features of patients used in this study.

## Methods

Unmodified DNA/RNA strands were obtained from commercial suppliers (IDT, Exiqon). Modified strands were prepared and characterized as described in [25].

Antibodies were provided by SSI, Denmark (anti-dsDNA, anti-β2-microglobulin monoclonal antibodies), or purchased from a commercial supplier (a-CL, polyclonal antibody, Immunovision, Catalog No. HCL-0200; anti-dsRNA, monoclonal antibody, Kerafast, Catalog No. ES2001). Plate coating was done following published procedure ([25]; data in S1 Appendix). Coating efficiency was confirmed by ELISA of 31 adult SLE samples (data in S1 Appendix). ELISA was carried out as described in [25]. Ss-DNA detected by ELISA, cut off >69, Inova Diagnostics, San Diego, CA, USA). Cut-off value for positivity was determined for each antigen as two standard deviations above the mean value for healthy controls [26–28] (Table B in S2 Appendix). For LS samples, an additional matched group of healthy controls was applied (Table C in S3 Appendix). The LS samples (n = 60) were derived from a well characterized clinical cohort, the National Registry of Childhood Onset Scleroderma housed at the University of Pittsburgh (Torok, PI). All sera were stored at– 20°C until being analyzed. The personal data of subjects was not disclosed for the project. Therefore no informed consent was needed for the serum samples. The study was approved by Dr. Sue Beers, IRB REN18010324 / PRO11060222.

### Data analysis

For the multivariate data analysis, we used the statistical modelling framework McGLM [29], where models are fitted under second moment assumptions (as opposed to fully specified distributional assumptions) using a scoring algorithm [29, 30] based on quasi-likelihood and estimating functions. Thus, a good compromise between robustness and efficiency is obtained. For the datasets with repeated measures structures induced by the patients, we used the well-known compound symmetry covariance structure to account for within-patient correlation. To reduce multicollinearity, we used stepwise selection of covariates based on variance inflation factors. Finally, to evaluate the fit of the models, we computed several goodness-of-fit measures.

## Results and discussion

### Antigens

Antigens were designed following our recently developed procedure [25]. In brief, a TC-rich sequence was expected to show the most effective binding to anti-DNA antibodies, and therefore was considered first (Table 1, D1). LNA were added to every fourth nucleotide in the sequence (23%), resulting in L1. Both single-stranded and double-stranded variants of the antigens were used (D1D and L1D). Next, the TC dinucleotide pair was changed to AT, giving D2. The rationale for this step was that we wanted to maintain a hydrogen bond formed by dT within the antigen sequence, while changing its position and the neighboring nucleotide. LNA were also incorporated in the resulting sequence, giving L2 and L2D. We also used mixmer sequences, D3, L3, and their duplexes, in this study. RNA variants of the DNA antigens were included in the series as natural and partially 2’OMe/LNA (R1–R3 and their variants). The control antigens C1 and C2 were adopted based on the literature. Rerefence C1 is a calf thymus dsDNA and C2 is a G-quadruplex forming ssDNA [31]. These molecules were expected to bind exclusively a-dsDNA/a-ssDNA.

**Table 1:** Antigen sequences and specificity to monoclonal (a-dsDNA/a-RNA, a-β2-micro-globulin), and polyclonal (a-CL) antibodies.

#	Sequence, 5’-3’	ss/ds	Modifi-cation	Antibody binding		
				a-dsDNA/ a-RNA	a-β2-micro-globulin	a-CL
	**DNA antigens**					
D1	5’-d(TC)_10_dT-3’	ss	-	+	+	++
D1D	5’-d(TC)_10_dT-3’: 3’-d(AG)_10_dA-5’	ds	-	+++	-	-
L1	5’-d(TCTC5MeC)_5_dT-3’	ss	LNA	-	-	-
L1D	5’-d(TCTC^5MeC^)5dT-3’: 3’-d(AG)_10_dA-5’	ds	LNA	++	-	-
D2	5’-d(AT)_10_dT-3’	ss	-	+	-	-
D2D	5’-d(AT)_10_dT-3’: 3’-d(TA)_10_dA-5’	ds	-	++	+	-
L2D	5’-d(ATAT^L^)_5_dT-3’: 3’-d(TA)_10_dA-5’	ds	LNA	++	-	-
D3	5’-d(ATCG)_5_dA-3’	ss	-	+	-	+
D3D	5’-d(ATGC)_5_dA-3’: 3’-d(TACG)_5_dT-5’	ds	-	++	-	-
L3D	5’-d(ATGC^5MeL^)_5_dA-3’: 3’-d(TACG)_5_dT-5’	ds	LNA	+	-	-
	**RNA antigens**					
R1	5’-r(UC)_10_rU-3’	ss	-	++	-	-
R1D	5’-r(UC)_10_rU-3’: 3’-d(AG)_10_dA-5’	ds	-	+++	-	+
L4	5’-r(UC)_10_rU-3’ with LNA	ss	LNA	-	+	-
L4D	5’-r(UC)_10_rU-3’: 3’-r(AG)_10_rA-5’ with LNA	ds	LNA	++	+	-
L4D-2	5’-r(UC)_10_rU-3’: 3’-r(AG)_10_rA-5’ with LNA	ds	LNA	+	+	-
R2D	5’-2’OMe(UC)_10_rU-3’: 3’-r(AG)_10_rA-5’	ds	2’-OMe	+	++	-
L5D	5’-2’OMe(UCUC^5MeL^)_5_rU-3’: 3’-r(AG)_10_rA-5’	ds	2’-OMe, LNA	+	++	-
R3	5’-r(AUGC)_5_rA-3’	ss	-	++	-	-
R3D	M5: 5’-r(AUGC)_5_rA-3’: 3’-r(UACG)_5_rU-5’	ds	-	+++	+	-
L6	M5: 5’-r(AUGC^5MeL^)_5_rA-3’	ss	LNA	+	+	-
L6D	M5: 5’-r(AUGC^5MeL^)_5_rA-3’: 3’-d(TACG)_5_dT-5’	ds	LNA	++	-	+
L6D-2	M5: 5’-r(AUGC^5MeL^)_5_rA-3’: 3’-r(UACG)_5_rU-5’	ds	LNA	+	-	-
	**Controls**					
C1	Calf thymus DNA	ds	-	++	+	+++
C2^25^	TTAGGGTTAGGGTTAGGGTTAGGGTTAG	ss	-	++	+	+++

B^L^ = LNA; C^5MeC^ = 5-methyl cytosine LNA. See the original absorbance values in the S2 Appendix.

The binding to autoimmune antibodies is a crucial parameter for antigens, which was first evaluated. We applied indirect ELISA with the antigens listed in Table 1 and the antibodies anti-dsDNA and anti-RNA as positive controls for DNA and RNA antigens, respectively; anti-β2-microglobulin and anti-cardiolipin (anti-CL) were employed as negative controls. All antibodies besides a-CL were monoclonal. The assay was carried out as follows: pre-diluted antibody was incubated with microtiter plates coated with corresponding antigens over 1.5 h at 37°C, known to be equilibrium conditions [21]. After washing, secondary antibody labeled with the histidine protein (HPr) enzyme was added and the incubation was maintained for an additional 1.5 h at 37°C. In the final step, 3,3',5,5'-tetramethylbenzidine (TMB) was added and upon its oxidation, the ultraviolet (UV) active product was detected by absorbance measurement. Each sample was analyzed in duplicate with a result deviation < 3%.

**Fig 2:**
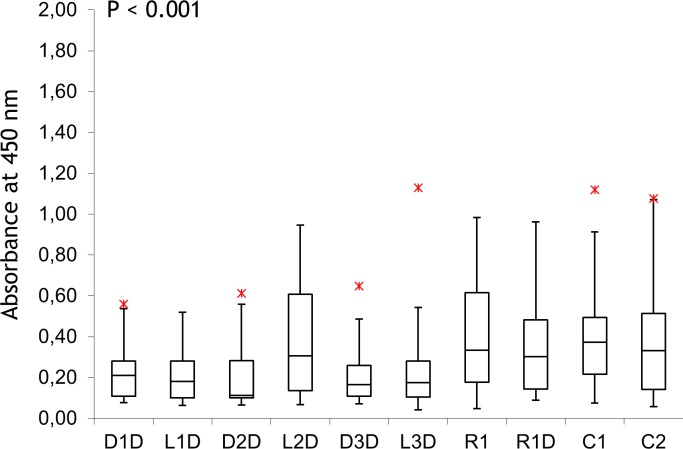
Analysis of new antigens in human sera. The data is presented in a modified box plot with observations outside the range [Q1 − 1.5 IQR, Q3 + 1.5 IQR] being considered outliers and identified using asterisks. Data points for each subject are means for three independent measurements with error < 3%. ELISA was carries out for unmatched healthy controls, n = 60 (see Methods for details). For absorbance values, see data in S2 Appendix.

Based on our results, we are able to draw three major conclusions. First, both the nucleotide content and chemical modifications have a major impact on the antigen recognition by antibody, see, e.g., D1D vs L2D, and D3D vs. L3D, Table 1. Effect of nucleotide content on recognition is well known for proteins and antibodies [24, 32]. However, the effect of chemical modification has not been investigated for a-DNAs in localized scleroderma before. Second, RNA variants might lack specificity as most of them effectively recognize negative controls. Third, single-strand antigens and C1–C2 show high binding to negative controls and to a-CL in particular (Fig 2). This result is not expected for C1-C2. However, it is known that the recognition of negatively charged cardiolipin by antibodies is mainly due to electrostatic interactions. Our data suggest that ssDNA, but not RNA, and large genomic CTD might interact with antibodies due to negative charge rather than by forming bonds via nucleotide-amino acid interactions. In turn, RNA are known to form advanced 3D structures [33]. This could contribute to their recognition by a-β2-mioglobulin.

### Studies of human samples

To test the antigens further, we used them in ELISA of an unmatched healthy cohort obtained from the Odense University Hospital, Denmark (n = 60; the samples were selected randomly; details are given in the S3 Appendix). As can be seen in Fig 2 below and in Table B in S2 Appendix, calf thymus DNA (C1) indicates 4 (5%) healthy controls as positives. This value is 4-fold lower for D1D confirming higher specificity. The reason for the higher binding by C1 could be due to the interactions with antibodies of no relevance to LS. It is interesting that the effect of LNA on the specificity is sequence dependent (e.g. D2D and D3D vs. L2D and L3D). However LNA increases the number of positives in healthy controls compared to unmodified DNA (e.g. 5 vs. 9 positive subjects detected by D2D vs. L2D, Table B in S2 Appendix).

Next, we collected a panel of human samples diagnosed with LS (see data in S3 Appendix for clinical and demographic details concerning the subjects). We aimed to study three key questions that have not previously been addressed: 1) the sequence specificity of LS-associated antibodies; 2) the potential to improve analytical and clinical specificity of the a-DNA assay using sequence-controlled antigens; and 3) the effects of the clinical features of LS subjects on anti-nucleic acid antibodies. The LS samples were 30 active/inactive disease pairs (totalling 60 data points, two for each patient) derived from a well characterized clinical cohort, the National Registry of Childhood Onset Scleroderma housed at the University of Pittsburgh (Torok, PI). Disease activity was evaluated following recommended criteria [24, 25]. During the treatment course, ANA patterns for LS patients were: 5 speckled, 2 homogenous, 1 nucleolar; ANA levels detected by clinical laboratory (HEp-2 assay, [7]) were: 1:80 (n = 2; speckled and nucleolar), 1:160 (n = 3, 2 speckled and 1 homogenous), 1:320 (n = 2, speckled and homogenous), 1:640 (n = 1, homogenous). At sampling, three LS subjects were ANA positive, with the levels 1:160 (n = 2) and 1:320 (n = 1), all speckled. As a control, we used our previously described pediatric SLE cohort (n = 27) [25].

**Fig 3:**
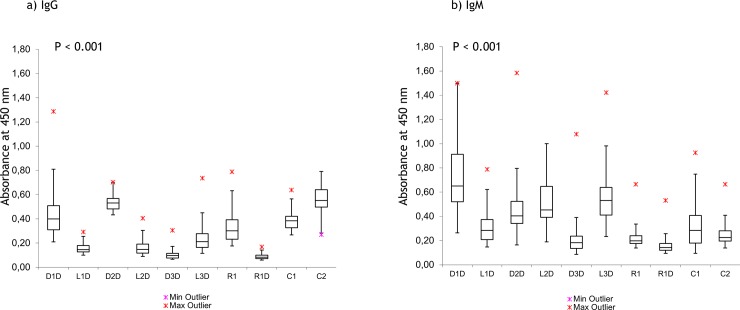
**Results of IgG (A) and IgM (B) ELISA assay for LS subjects (n = 30) using DNA and LNA/DNA antigens.** Both figures are modified box plots, where observations outside the range [Q1 − 1.5 IQR, Q3 + 1.5 IQR] are considered outliers and identified using asterisks. Data points for each subject are means for three independent measurements with error < 3%. For absorbance values, see data in S2 Appendix.

For this study, we selected the antigens showing good specificity as evaluated in the antibody test, a-CL and healthy control analysis (Table 1, Fig 2), along with C1–C2. The assay was carried out as described below, using patient samples in the primary incubation step. The results of the ELISA testing are shown in Fig 3. For LS, we analyzed IgG and IgM in separate ELISA tests. Anti-DNA of the IgG isotype is the most common in LS [13–16]. Nevertheless, we included IgM a-DNA and IgM a-RNA in this study to evaluate the potential effect of LNA and DNA/RNA sequences on recognition of a different class of anti-DNA.

In the IgG profile (Fig 3A), the highest number of positive samples is shown for the antigens D1D, D2D, and the control C2, whereas the mixmer D3D shows nearly no positive signals. Similar to SLE, the polyclonal antibodies to nucleic acids in LS are indeed sequence specific, and most effectively recognize single- and double-stranded [dT] DNA. Interestingly, the control C1 exhibits less binding than other antigens, e.g., vs. C2, which displays high binding.

Another striking finding is that the incorporation of LNA monomers in the 21-mer antigens has a highly dramatic influence on antibody recognition. For D1D and D2D, the signal decreases 4–8-fold upon LNA incorporation. However, for D3D and L3D, the effect of LNA on binding levels is the opposite.

For IgM, the binding levels differed from IgG (Fig 3B compared to Fig 3A; data in S2 Appendix). D1D still showed the highest signal; however, the influence of LNA on the antibody levels changed. Specifically, the signal decreased for L1D compared to D1D, and increased for L2D–L3D vs. LNA-free D2D and D3D. Both C1 and C2 exhibited a low signal. For RNA antigens, the difference in binding levels was as dramatic as in the IgG test.

We compared the binding levels for LS across novel antigens with another disease, pSLE (n = 27) (see data in S3 Appendix for subject information). Compared to LS, pSLE have higher binding levels for all antigens, with a lower sensitivity to a particular sequence. This could be caused by high disease activity in the pSLE cohort (Table A in S3 Appendix). Nevertheless, the influence of LNA was similar for LS and pSLE: binding to L1D–L2D decreased compared to the DNA analogues D1D–D2D, and increased for the mixmer L3D vs. DNA D3D. Lower binding to G-quadruplex C2 was observed and a high level of antibodies in pSLE recognized CTD C1.

Using D1D, 30% (n = 18) LS samples were positive. This value was 6-fold higher than for ANA determined by clinical laboratory (5% positive), and 1.5-fold higher than AHA (20% positive; Table A in S3 Appendix). Notably, in the control assays C1-C2 no positive samples were detected (Table C in S2 Appendix).

### Statistical analyses

For the multivariate data analysis, we adopted an extension of generalized linear models called multivariate covariance generalized linear models (McGLMs) as implemented in the R [27] package mcglm [28], which is a very general and comprehensive framework proposed by Bonat and Jørgensen [29]. In this framework, the models are fitted using an estimating function approach based on second moment assumptions only, which provides some robustness against misspecification of the models. In both models, the variables D1D, L1D, D2D, L2D, D3D, L3D, C1, and C2 serve as response variables, while the variables Time in Treatment, mLoSSI, LoSSI, PGAACT, and PGADAMG serve as covariates for all response variables. In order to reduce multicollinearity, we used stepwise selection of covariates based on variance inflation factors. Table Q in S4 Appendix was used to assess multicollinearity among covariates in the final models. In the model fitted to the IgG LS dataset, we used the log link function and the Tweedie variance function (where the power parameter is estimated) for the response variables D1D, L2D, D3D, and L3D, and the identity link function and the constant variance function for the response variables L1D, D2D, C1, and C2. In the model fitted to the IgM LS dataset, we used the log link function and the Tweedie variance function (where the power parameter is estimated) for the response variables D1D, L1D, L2D, D3D, L3D, C1, and C2, and the identity link function and the constant variance function for the response variable D2D. Both datasets have a repeated measures structure which may induce some correlation between observations on the same patient. Thus, it seemed reasonable to account for within-patient correlation induced by the repeated measurements using the well-known compound symmetry covariance structure, defined by a linear combination of an identity matrix and a matrix of 1s. However, since the repeated measures structure was not significant for any response variable in the model fitted to the IgG LS dataset, we assumed independent observations. In the model fitted to the IgM dataset, the repeated measures structure was significant for the response variable D1D only, but since it had no impact on the final results in terms of significance of the covariates, we assumed again independent observations. Finally, to evaluate the fit of the models, we computed several goodness-of-fit measures. In general, we observe that the multivariate models provide a better fit to the data than the univariate models. Moreover, to obtain a more parsimonious model for the IgG LS dataset, we used a stepwise regression procedure with Bayesian information criterion (BIC).

The main question we would like to address in this work is if there are specific polyclonal antibodies where the level of binding correlates with other disease activity measures or correlates with several disease activity measures used together. To answer on this, we studied the effects of clinical features of the LS subjects on the detected antibody titers. We use the models to make inferences about the effects of clinical features from IgG and IgM datasets. In particular we focused on effects of disease indices of activity on antibodies to new antigens: mLoSSI and PGAACT, and indices of disease damage: LoSDI and PGADAMG. Time in treatment (treatment duration to time of blood sample) was included as well.

According to our analysis, IgG and IgM datasets varied (Table 2 and data in S4 Appendix). For IgG, mLoSSI, which is characteristics of active LS, had significant effect on G-quadruplex forming C2 [31]. The localized scleroderma skin damage index LoSDI, but not PGADAMG, had an effect on several biomarkers: a-D1D, a-L2D, a-L3D, and C1. This indicates a potential linking for a-DNA to the biological status of disease state in LS which has not been reported before. On the contrary, PGADAMG (disease damage index) reflects cumulative disease but not necessarily biologic activity [9, 20]. Therefore the absence of a correlation for the PGADAMG with the a-DNA additionally supports the hypothesis on their biological role in LS. Interestingly, the model suggests that LoSDI does not have an effect on the antibodies to control C2. It is also interesting that the effects changed for the analogues a-D1D vs. a-L1D, a-D2D vs. a-L2D, and a-D3D vs. a-L3D.

**Table 2:** Results of statistical analyses of IgG ELISA data. Effects at a significance level of 0.05 (p<0.05) are shown in red.

Biomarker/ Clinical features	Disease activity indexes		Disease damage indexes		Time in treatment
	mLoSSI	PGAACT	LoSDI	PGADAMG	
**a-D1D**					
**a-L1D**					
**a-D2D**					
**a-L2D**					
**a-D3D**					
**a-L3D**					
**C1**					
**C2**					

Time in treatment had an effect exclusively on a-D1D and a-D3D (Table 2). This is an interesting observation given the complete lack of this effect in the antibodies to controls and other synthetic antigens used in this work. No other tested parameters showed significant effects on the antibodies to either synthetic antigens or controls (data in S4 Appendix). As to IgM, LoSDI and time in treatment had an effect on most variables including C1-C2; little to no difference was seen for LNA vs. DNA analogues (data in S4 Appendix).

Finally, we compared the quantitative characteristics of our tests in the detection of antibodies related to LS with the results for novel antigens. The results of ANA and anti-ssDNA testing in LS are typically reported as either the dilution of serum that tests positive or the degree of positivity measured by the testing procedure (staining pattern) [34, 35]. Having performed a sera dilution study, we concluded that the novel antigens show up to 10-fold higher analytical sensitivity over the control C1 and commercial anti-ssDNA kit (data in S3 Appendix), most likely due to the high purity (> 90%) of the former.

## Conclusions

In conclusion, we have designed and tested new synthetic nucleic acids as antigens for the diagnosis and characterization of localized scleroderma (LS). Besides showing higher analytical specificity for the detection of LS-associated antibodies, our work implies biological relevance of these biomarkers in the LS. According to our results, synthetic TC-rich DNA is the most efficient antigen for the detection of active LS disease, albeit as a double-strand variant only. Several other antigens also show effects of clinical parameters among LS patients on IgG antibody levels. This is in contrast to the rather poor performance of the DNA antigens, especially when studying healthy controls.

Our studies suggest that single-stranded DNA and RNA impurities could contribute to the false positive results when detecting antibodies to dsDNA. Therefore, their presence in DNA antigens has to be controlled with care. Moreover, LNA is often applied in aptamer development. Our work shows that LNA incorporation affects antibody binding and therefore requires careful controls.

Based on our results, we believe that rationally designed oligonucleotides could aid in the discovery of a new generation of biomarkers for the specific diagnosis and point-of-care monitoring of LS and other autoimmune diseases that involve the development of antibodies to nucleic acids. This includes, but not limited to, systemic lupus erythematosus and autoimmune hepatitis [17, 36, 37].

## Supporting information

S1 Appendix ELISA plate preparation and coating control. (DOCX)

S2 Appendix ELISA results for the antibody tests. (DOCX)

S3 Appendix Characteristics of subjects used in this study and assay establishment. (DOCX)

S4 Appendix Statistical analyses. (DOCX)
