# Supplementary material for: Detection of autoimmune antibodies in localized scleroderma by synthetic oligonucleotide antigens
Source: PLoS One. 2018 Apr 11;13(4):e0195381. doi: 10.1371/journal.pone.0195381 (PMC5895021; doi:10.1371/journal.pone.0195381)
Supplement: S1 Appendix — (DOCX) [file pone.0195381.s001.docx]

# Abbreviations

SLE - systemic lupus erythematosus

SLEDAI – systemic lupus erythematosus disease activity index

IgG – immunoglobulin class G

IgM – immunoglobulin class M

ELISA – enzyme-linked immunosorbent assay

HPR – horseradish peroxidase

ESR - [Erythrocyte Sedimentation Rate](https://labtestsonline.org/understanding/analytes/esr/tab/test/)

PO/IV steroids – peroral/intravenous steroids

HCQ - hydroxyquinoline

IS – immunosuppressive drug

NSAID - non steroidal anti-inflammatory drug

# Plate coating and ELISA procedure^[[1]](#footnote-1)^

Maxisorb 96 well plates (NUNC Thermofisher) were coated with ss/ds antigens at concentration 2 µg/mL in 1X PBS overnight (RT; 150 µl/well). After washing with 1X PT (2 × 300 µl/well, PT: 50 µl Tween-20 in 1 L 1X PBS), the plates were blocked with 1X PTB (1 h, 37 ° C; 100 µl/well, PTB: 20 g BSA, 50 µl Tween-20 in 1 L 1X PBS). Incubation with plasma at desired dilution was performed at 37 °C for 1.5 h using diluent: 2 g BSA, 50 µl Tween-20 in 1 L 1X PBS (100 µl/well). This was followed by washing (2 × 300 µl 1X PBS) and incubation with HPR-labelled secondary antibody for 1.5 h at 37 ° C using same diluent and dilution of the secondary antibody provided by supplier (HPR-conjugated a-aIgG or a-aIgM; Sigma). Subsequent washing (2 × 300 µl PT) and incubation with freshly prepared TMB-H_2_O_2_ solution (Sigma; 100 µl/well) was followed by adding a stop solution (1M H_2_SO_4_; 50 µl/well) and reading resulting absorbance values at 450 nm on Magellan Tecan microplate reader. Linear range for each antigen was determined via testing series of control dilutions (randomly selected 5 LS, 5 adult SLE and 5 healthy controls in dilutions 1:50 to 1:2000). According to the results plasma dilutions 1:100 - 1:500 were within linear range of the assay for each antigen (R^2^ > 0.95).

**Bradford assay.** Prior to ELISA, total amount of protein in each sample was estimated by Bradford method using standard curve of BSA control at known concentration (BioRad). In a maxisorb 96 well plate controls (BSA standard samples at concentrations 2 mg/mL, 1 mg/mL, 0.5 mg/mL and 0.1 mg/mL) and plasma sample were mixed with a Bradford reagent following manufacturer’s protocol (BioRad). Plasma samples were used in dilution 1:100. Resulting absorbances at 595 nm were measured on Magellan Tecan microplate reader. Total amount of protein was calculated using standard curve.

**Table A. Demographic and clinical characteristics of adult SLE subjects.**

| **Variable** | **Adult SLE (n=31),**  **Stanford** |
| --- | --- |
| Female/male | 27/4 |
| Hispanic (%) | 6 (19) |
| Asian/Pacific Islander (%) | 6 (19) |
| Non-Hispanic Caucasian (%) | 6 (19) |
| African-American (%) | 4 (13) |
| Age at diagnosis, median (range) years | 24 (2-44) |
| Age at sample collection, median, (range) years | 38 (20-68) |
| dsDNA antibody positive patients at onset – *Crithidia assay* (%) | 12 (39) |
| Class III/IV lupus nephritis-biopsy proven† (%) | nd |
| Mean (range) SLEDAI | 3.2 (0-8) |
| Mean (range) ESR | nd |
| Mean (range) C3 complement | 91.4 (26-141) |
| Treatment at sample*: |  |
| PO steroids (%) | 20 (64.5) |
| IV steroids (%) | 0 (0) |
| HCQ (%) | 22 (71) |
| Other IS (%) | 18 (58) |
| Daily NSAID (%) | 6 (19) |
| None | 0 (0) |

nd = no data. Other medication: prednisone, HCQ, IV solumedrol, cellcept, ranitidine, piroxicam.

Absorbance at 450 nm

Synthetic antigens

**Fig A.** **Box-and-whisker plot with outliers for the results of ELISA performed for adult SLE samples (n=31).** The arms on each boxplot are values Q1 – 1.5 × IQR and Q3 + 1.5 × IQR. Data points for each subject are means for three independent measurements. Cut off values for positivity were determined as 2-fold levels above the mean value for healthy controls (n=60) tested on each antigen, and were: 0.650 (L1D, L2D and L3D) and 0.850 (D1D, D2D and D3D).

1. Samuelsen SV, Solov'yov IA, Balboni IM, [Mellins E](https://www.ncbi.nlm.nih.gov/pubmed/?term=Mellins%20E%5BAuthor%5D&cauthor=true&cauthor_uid=27775006), Nielsen CT, Heegaard NH and Astakhova K. Synthetic oligonucleotide antigens modified with locked nucleic acids detect disease specific antibodies. Sci Rep*.* 2016; 6: 35827. [↑](#footnote-ref-1)
