# Supplementary material for: Detection of autoimmune antibodies in localized scleroderma by synthetic oligonucleotide antigens
Source: PLoS One. 2018 Apr 11;13(4):e0195381. doi: 10.1371/journal.pone.0195381 (PMC5895021; doi:10.1371/journal.pone.0195381)
Supplement: S2 Appendix — (DOCX) [file pone.0195381.s002.docx]

**Table A.** **ELISA results for monoclonal and polyclonal antibodies detected by novel antigens and controls.***

| # | Sequence, 5’-3’ | ss/ds | Modifi-cation | Absorbance at 450 nm: | | |
| --- | --- | --- | --- | --- | --- | --- |
|  |  |  |  | a-dsDNA/  a-RNA | a-β2-micro-globulin | a-CL |
|  | **DNA antigens** |  |  |  |  |  |
| D1 | 5’-d(TC)_10_dT-3’ | ss | - | 0.62 | 0.59 | 0.81 |
| D1D | 5’-d(TC)_10_dT-3’: 3’-d(AG)_10_dA-5’ | ds | - | 1.01 | 0.18 | 0.23 |
| L1 | 5’-d(TCTC^5MeC^)_5_dT-3’ | ss | LNA | 0.17 | 0.21 | 0.11 |
| L1D | 5’-d(TCTC^5MeC^)_5_dT-3’: 3’-d(AG)_10_dA-5’ | ds | LNA | 0.76 | 0.24 | 0.23 |
| D2 | 5’-d(AT)_10_dT-3’ | ss | - | 0.55 | 0.32 | 0.16 |
| D2D | 5’-d(AT)_10_dT-3’: 3’-d(TA)_10_dA-5’ | ds | - | 0.73 | 0.57 | 0.42 |
| L2D | 5’-d(ATAT^L^)_5_dT-3’: 3’-d(TA)_10_dA-5’ | ds | LNA | 0.76 | 0.18 | 0.34 |
| D3 | 5’-d(ATCG)_5_dA-3’ | ss | - | 0.59 | 0.11 | 0.57 |
| D3D | 5’-d(ATGC)_5_dA-3’: 3’-d(TACG)_5_dT-5’ | ds | - | 0.62 | 0.19 | 0.12 |
| L3D | 5’-d(ATGC^5MeL^)_5_dA-3’: 3’-d(TACG)_5_dT-5’ | ds | LNA | 0.54 | 0.21 | 0.15 |
|  | **RNA antigens** |  |  |  |  |  |
| R1 | 5’-r(UC)_10_rU-3’ | ss | - | 0.77 | 0.23 | 0.20 |
| R1D | 5’-r(UC)_10_rU-3’: 3’-d(AG)_10_dA-5’ | ds | - | 1.12 | 0.22 | 0.59 |
| L4 | 5’-r(UC)_10_rU-3’ with LNA | ss | LNA | 0.32 | 0.64 | 0.23 |
| L4D | 5’-r(UC)_10_rU-3’: 3’-r(AG)_10_rA-5’ with LNA | ds | LNA | 0.80 | 0.57 | 0.20 |
| L4D-2 | 5’-r(UC)_10_rU-3’: 3’-r(AG)_10_rA-5’ with LNA | ds | LNA | 0.53 | 0.71 | 0.14 |
| R2D | 5’-2’OMe(UC)_10_rU-3’: 3’-r(AG)_10_rA-5’ | ds | 2’-OMe | 0.62 | 0.84 | 0.17 |
| L5D | 5’-2’OMe(UCUC^5MeL^)_5_rU-3’: 3’-r(AG)_10_rA-5’ | ds | 2’-OMe, LNA | 0.57 | 0.82 | 0.33 |
| R3 | 5’-r(AUGC)_5_rA-3’ | ss | - | 0.83 | 0.34 | 0.23 |
| R3D | M5: 5’-r(AUGC)_5_rA-3’: 3’-r(UACG)_5_rU-5’ | ds | - | 1.42 | 0.61 | 0.31 |
| L6 | M5: 5’-r(AUGC^5MeL^)_5_rA-3’ | ss | LNA | 0.58 | 0.70 | 0.28 |
| L6D | M5: 5’-r(AUGC^5MeL^)_5_rA-3’: 3’-d(TACG)_5_dT-5’ | ds | LNA | 0.81 | 0.26 | 0.55 |
| L6D-2 | M5: 5’-r(AUGC^5MeL^)_5_rA-3’: 3’-r(UACG)_5_rU-5’ | ds | LNA | 0.54 | 0.21 | 0.14 |
|  | **Controls** |  |  |  |  |  |
| C1 | Calf thymus DNA | ds | - | 0.81 | 0.55 | 1.56 |
| C2^25^ | TTAGGGTTAGGGTTAGGGTTAGGGTTAG | ss | - | 0.88 | 0.70 | 1.11 |

B^L^ = LNA; C^5MeC^ = 5-methyl cytosine LNA. Cut off values were established using healthy controls as 2 standard deviations (2 SD), 3 SD and 4 SD above the mean healthy control value for weak (+), medium range (++) and strong positive (+++) results. The actual absorbance ranges were 0.53-0.71 (+), 0.71-0.89 (++) and > 0.89 (+++). Each value is a mean for triplicate measurement with a deviation between results < 5%.

**Table B.** **IgG** **ELISA results for healthy controls.**

| #/A_450_ | **Antigen:**  D1D | | L1D | D2D | L2D | D3D | L3D | R1 | R1D | C1 | C2 |
| --- | --- | --- | --- | --- | --- | --- | --- | --- | --- | --- | --- |
| 1 | 0.05 | | 0.12 | 0.40 | 0.45 | 0.09 | 0.11 | 0.19 | 0.23 | 0.31 | 0.13 |
| 2 | 0.23 | | 0.18 | 0.28 | 0.61 | 0.16 | 0.10 | 0.22 | 0.96 | 0.56 | 0.85 |
| 3 | 0.21 | | 0.28 | 0.13 | 0.90 | 0.11 | 0.04 | 0.62 | 0.21 | 0.11 | 0.13 |
| 4 | 0.09 | | 0.23 | 0.07 | 0.07 | 0.23 | 0.10 | 0.11 | 0.10 | 0.34 | 0.16 |
| 5 | 0.08 | | 0.09 | 0.14 | 0.11 | 0.07 | 0.15 | 0.12 | 0.10 | 0.78 | 0.09 |
| 6 | 0.11 | | 0.10 | 0.10 | 0.31 | 0.21 | 0.27 | 0.10 | 0.12 | 0.39 | 0.14 |
| 7 | 0.14 | | 0.08 | 0.07 | 0.22 | 0.08 | 0.12 | 0.10 | 0.09 | 0.22 | 0.25 |
| 8 | 0.10 | | 0.08 | 0.22 | 0.07 | 0.09 | 0.08 | 0.08 | 0.09 | 0.44 | 0.08 |
| 9 | 0.08 | | 0.10 | 0.61 | 0.45 | 0.32 | 0.28 | 0.49 | 0.64 | 0.56 | 0.30 |
| 10 | 0.11 | | 0.52 | 0.59 | 0.81 | 0.41 | 0.26 | 0.86 | 0.89 | 0.32 | 0.83 |
| 11 | 0.22 | | 0.10 | 0.45 | 0.92 | 0.14 | 0.05 | 0.88 | 0.48 | 0.30 | 0.36 |
| 12 | 0.34 | | 0.41 | 0.42 | 0.35 | 0.27 | 0.42 | 0.30 | 0.30 | 0.34 | 0.45 |
| 13 | 0.33 | | 0.38 | 0.29 | 0.29 | 0.28 | 0.45 | 0.40 | 0.40 | 0.39 | 0.33 |
| 14 | 0.12 | | 0.35 | 0.10 | 0.64 | 0.55 | 0.34 | 0.45 | 0.41 | 0.31 | 0.42 |
| 15 | 0.08 | | 0.31 | 0.30 | 0.36 | 0.15 | 0.23 | 0.43 | 0.50 | 0.40 | 0.51 |
| 16 | 0.34 | | 0.23 | 0.38 | 0.37 | 0.27 | 0.28 | 0.33 | 0.35 | 0.41 | 0.35 |
| 17 | 0.34 | | 0.11 | 0.10 | 0.85 | 0.34 | 0.06 | 0.56 | 0.40 | 0.23 | 0.40 |
| 18 | 0.40 | | 0.10 | 0.10 | 0.13 | 0.11 | 0.09 | 0.70 | 0.52 | 0.76 | 1.08 |
| 19 | 0.40 | | 0.10 | 0.45 | 0.07 | 0.18 | 0.22 | 0.45 | 0.92 | 0.45 | 0.52 |
| 20 | 0.50 | | 0.24 | 0.06 | 0.51 | 0.19 | 0.15 | 0.05 | 0.50 | 0.98 | 0.06 |
| 21 | 0.27 | | 0.11 | 0.10 | 0.32 | 0.49 | 0.28 | 0.98 | 0.37 | 1.12 | 0.75 |
| 22 | 0.28 | | 0.32 | 0.10 | 0.12 | 0.08 | 0.13 | 0.52 | 0.38 | 0.32 | 0.67 |
| 23 | 0.22 | | 0.37 | 0.10 | 0.16 | 0.10 | 0.27 | 0.62 | 0.42 | 0.37 | 0.28 |
| 24 | 0.56 | | 0.49 | 0.10 | 0.33 | 0.13 | 0.54 | 0.51 | 0.56 | 0.49 | 0.47 |
| 25 | 0.42 | | 0.38 | 0.10 | 0.12 | 0.25 | 0.18 | 0.67 | 0.42 | 0.38 | 0.74 |
| 26 | 0.21 | | 0.10 | 0.48 | 0.16 | 0.09 | 0.15 | 0.21 | 0.14 | 0.21 | 0.29 |
| 27 | 0.15 | | 0.23 | 0.10 | 0.26 | 0.19 | 0.42 | 0.68 | 0.32 | 0.68 | 0.58 |
| 28 | 0.27 | | 0.28 | 0.12 | 0.67 | 0.23 | 1.13 | 0.88 | 0.22 | 0.88 | 0.49 |
| 29 | 0.34 | | 0.06 | 0.08 | 0.28 | 0.17 | 0.23 | 0.33 | 0.22 | 0.17 | 0.11 |
| 30 | 0.11 | | 0.09 | 0.10 | 0.66 | 0.31 | 1.11 | 0.33 | 0.52 | 0.48 | 0.22 |
| 31 | 0.18 | | 0.22 | 0.18 | 0.94 | 0.14 | 0.13 | 0.63 | 0.14 | 0.41 | 0.23 |
| 32 | 0.19 | | 0.15 | 0.11 | 0.10 | 0.26 | 0.18 | 0.18 | 0.15 | 0.12 | 0.16 |
| 33 | 0.20 | | 0.28 | 0.24 | 0.16 | 0.18 | 0.44 | 0.22 | 0.25 | 0.22 | 0.18 |
| 34 | 0.08 | | 0.13 | 0.12 | 0.95 | 0.65 | 1.08 | 0.28 | 0.23 | 0.13 | 0.37 |
| 35 | 0.22 | | 0.14 | 0.13 | 0.14 | 0.12 | 0.17 | 0.19 | 0.14 | 0.14 | 0.64 |
| 36 | 0.26 | | 0.11 | 0.11 | 0.12 | 0.10 | 0.08 | 0.09 | 0.16 | 0.18 | 0.08 |
| 37 | 0.22 | | 0.35 | 0.40 | 0.45 | 0.09 | 0.11 | 0.19 | 0.23 | 0.77 | 0.13 |
| 38 | 0.13 | | 0.18 | 0.28 | 0.61 | 0.16 | 0.10 | 0.43 | 0.96 | 0.16 | 0.85 |
| 39 | 0.21 | | 0.28 | 0.13 | 0.90 | 0.11 | 0.14 | 0.62 | 0.21 | 0.10 | 0.13 |
| 40 | 0.09 | | 0.11 | 0.15 | 0.07 | 0.04 | 0.10 | 0.08 | 0.10 | 0.07 | 0.14 |
| 41 | 0.08 | | 0.21 | 0.09 | 0.08 | 0.07 | 0.15 | 0.12 | 0.10 | 0.34 | 0.09 |
| 42 | 0.11 | | 0.14 | 0.13 | 0.31 | 0.21 | 0.27 | 0.10 | 0.12 | 0.55 | 0.14 |
| 43 | 0.14 | | 0.17 | 0.07 | 0.09 | 0.08 | 0.12 | 0.10 | 0.21 | 0.08 | 0.25 |
| 44 | 0.10 | | 0.09 | 0.12 | 0.04 | 0.11 | 0.28 | 0.15 | 0.17 | 0.22 | 0.08 |
| 45 | 0.08 | | 0.10 | 0.61 | 0.61 | 0.32 | 0.17 | 0.49 | 0.64 | 0.34 | 0.32 |
| 46 | 0.11 | | 0.52 | 0.59 | 0.81 | 0.41 | 0.26 | 0.86 | 0.89 | 0.55 | 0.83 |
| 47 | 0.22 | | 0.10 | 0.45 | 0.92 | 0.38 | 0.05 | 0.88 | 0.48 | 0.30 | 0.33 |
| 48 | 0.34 | | 0.41 | 0.42 | 0.35 | 0.27 | 0.42 | 0.30 | 0.30 | 0.34 | 0.45 |
| 49 | 0.33 | | 0.38 | 0.29 | 0.29 | 0.28 | 0.42 | 0.40 | 0.40 | 0.66 | 0.30 |
| 50 | 0.12 | | 0.35 | 0.10 | 0.64 | 0.55 | 0.59 | 0.61 | 0.41 | 0.31 | 0.41 |
| 51 | 0.08 | | 0.31 | 0.30 | 0.36 | 0.15 | 0.23 | 0.43 | 0.50 | 0.40 | 0.51 |
| 52 | 0.34 | | 0.35 | 0.38 | 0.37 | 0.27 | 0.28 | 0.33 | 0.35 | 0.41 | 0.35 |
| 53 | 0.34 | | 0.11 | 0.10 | 0.85 | 0.34 | 0.06 | 0.56 | 0.40 | 0.56 | 0.44 |
| 54 | 0.32 | | 0.10 | 0.13 | 0.13 | 0.11 | 0.09 | 0.70 | 0.52 | 0.54 | 1.08 |
| 55 | 0.21 | | 0.12 | 0.45 | 0.07 | 0.18 | 0.22 | 0.45 | 0.92 | 0.87 | 0.52 |
| 56 | 0.45 | | 0.24 | 0.06 | 0.51 | 0.19 | 0.15 | 0.07 | 0.50 | 0.24 | 0.06 |
| 57 | 0.37 | | 0.42 | 0.13 | 0.32 | 0.49 | 0.28 | 0.98 | 0.37 | 0.42 | 0.78 |
| 58 | 0.38 | | 0.31 | 0.14 | 0.12 | 0.08 | 0.13 | 0.89 | 0.38 | 0.32 | 0.60 |
| 59 | 0.42 | | 0.37 | 0.09 | 0.16 | 0.10 | 0.21 | 0.62 | 0.42 | 0.37 | 0.32 |
| 60 | 0.34 | | 0.49 | 0.10 | 0.33 | 0.13 | 0.51 | 0.51 | 0.56 | 0.49 | 0.48 |
|  |  | |  |  |  |  |  |  |  |  |  |
| 61 | 0.38 | | 0.31 | 0.08 | 0.26 | 0.10 | 0.20 | 0.30 | 0.24 | 0.14 | 1.08 |
| 62 | 0.48 | | 0.08 | 0.16 | 0.22 | 0.09 | 0.26 | 0.31 | 0.26 | 0.38 | 0.77 |
| 63 | 0.20 | | 0.09 | 0.08 | 0.32 | 0.13 | 0.24 | 0.16 | 0.09 | 0.29 | 0.60 |
| 64 | 0.46 | | 0.18 | 0.06 | 0.12 | 0.18 | 0.31 | 0.26 | 0.14 | 0.43 | 0.24 |
| 65 | 0.27 | | 0.12 | 0.10 | 0.29 | 0.13 | 0.27 | 0.21 | 0.10 | 0.29 | 0.30 |
| 66 | 0.21 | | 0.11 | 0.08 | 0.44 | 0.28 | 0.22 | 0.16 | 0.38 | 0.56 | 0.22 |
| 67 | 0.25 | | 0.12 | 0.06 | 0.28 | 0.16 | 0.14 | 0.19 | 0.09 | 1.09 | 0.23 |
| 68 | 0.21 | | 0.16 | 0.27 | 0.12 | 0.16 | 0.14 | 0.16 | 0.12 | 0.81 | 0.52 |
| 69 | 0.19 | | 0.08 | 0.05 | 0.40 | 0.12 | 0.13 | 0.15 | 0.06 | 0.10 | 0.79 |
| 70 | 0.41 | | 0.22 | 0.08 | 0.21 | 0.17 | 0.06 | 0.32 | 0.27 | 0.54 | 0.33 |
| 71 | 0.05 | | 0.02 | 0.06 | 0.12 | 0.04 | 0.07 | 0.14 | 0.11 | 0.18 | 0.70 |
| 72 | 0.38 | | 0.31 | 0.18 | 0.12 | 0.12 | 0.21 | 0.30 | 0.24 | 0.67 | 0.88 |
| 73 | 0.48 | | 0.08 | 0.06 | 0.22 | 0.19 | 0.21 | 0.37 | 0.06 | 0.95 | 0.17 |
| 74 | 0.20 | | 0.09 | 0.10 | 023 | 0.08 | 0.37 | 0.16 | 0.07 | 1.24 | 0.20 |
| 75 | 0.46 | | 0.18 | 0.09 | 0.31 | 0.19 | 0.30 | 0.36 | 0.14 | 0.17 | 0.14 |
| **BLANK** | | 0.026 | 0.011 | 0.021 | 0.012 | 0.025 | 0.008 | 0.009 | 0.016 | 0.014 | 0.007 |

*Blank is the signal obtained using incubation buffer instead of serum.

Cut off values for positivity (antigen): 0.50 (D1D), 0.47 (L1D), 0.51 (D2D), 0.68 (L2D), 0.46 (D3D), 0.68 (L3D), 0.89 (R1), 0.80 (R1D), 0.95 (C1), 0.94 (C2).

**Table C. IgG** **ELISA results for LS.**

| #/A_450_ | **Antigen:**  D1D | | L1D | D2D | L2D | D3D | L3D | R1 | R1D | C1 | C2 |
| --- | --- | --- | --- | --- | --- | --- | --- | --- | --- | --- | --- |
| 1 | 0.33 | | 0.13 | 0.58 | 0.11 | 0.09 | 0.22 | 0.38 | 0.07 | 0.41 | 0.71 |
| 2 | 0.31 | | 0.13 | 0.60 | 0.12 | 0.08 | 0.20 | 0.32 | 0.05 | 0.41 | 0.65 |
| 3 | 0.27 | | 0.14 | 0.64 | 0.10 | 0.07 | 0.16 | 0.22 | 0.08 | 0.42 | 0.56 |
| 4 | 0.21 | | 0.15 | 0.59 | 0.13 | 0.07 | 0.18 | 0.18 | 0.06 | 0.35 | 0.49 |
| 5 | 0.44 | | 0.12 | 0.56 | 0.17 | 0.07 | 0.31 | 0.44 | 0.04 | 0.27 | 0.55 |
| 6 | 0.58 | | 0.29 | 0.44 | 0.10 | 0.10 | 0.18 | 0.57 | 0.11 | 0.42 | 0.40 |
| 7 | 0.37 | | 0.13 | 0.54 | 0.10 | 0.08 | 0.21 | 0.34 | 0.18 | 0.30 | 0.59 |
| 8 | 0.32 | | 0.13 | 0.38 | 0.09 | 0.10 | 0.16 | 0.27 | 0.08 | 0.30 | 0.42 |
| 9 | 0.27 | | 0.10 | 0.40 | 0.29 | 0.07 | 0.47 | 0.22 | 0.13 | 0.34 | 0.48 |
| 10 | 0.65 | | 0.18 | 0.53 | 0.12 | 0.16 | 0.21 | 0.46 | 0.13 | 0.45 | 0.79 |
| 11 | 0.34 | | 0.15 | 0.45 | 0.24 | 0.08 | 0.31 | 0.28 | 0.10 | 0.37 | 0.72 |
| 12 | 0.43 | | 0.23 | 0.49 | 0.11 | 0.14 | 0.12 | 0.39 | 0.12 | 0.42 | 0.58 |
| 13 | 0.32 | | 0.13 | 0.61 | 0.12 | 0.08 | 0.19 | 0.18 | 0.06 | 0.33 | 0.53 |
| 14 | 0.37 | | 0.11 | 0.55 | 0.10 | 0.09 | 0.16 | 0.39 | 0.09 | 0.38 | 0.63 |
| 15 | 0.32 | | 0.13 | 0.60 | 0.10 | 0.07 | 0.15 | 0.31 | 0.07 | 0.29 | 0.54 |
| 16 | 0.29 | | 0.23 | 0.55 | 0.17 | 0.08 | 0.16 | 0.27 | 0.06 | 0.29 | 0.40 |
| 17 | 0.35 | | 0.12 | 0.47 | 0.17 | 0.07 | 0.30 | 0.35 | 0.07 | 0.28 | 0.55 |
| 18 | 0.77 | | 0.18 | 0.46 | 0.22 | 0.15 | 0.28 | 0.79 | 0.10 | 0.45 | 0.68 |
| 19 | 0.53 | | 0.19 | 0.49 | 0.14 | 0.12 | 0.24 | 0.46 | 0.10 | 0.40 | 0.55 |
| 20 | 0.44 | | 0.18 | 0.50 | 0.13 | 0.10 | 0.25 | 0.42 | 0.09 | 0.40 | 0.44 |
| 21 | 0.42 | | 0.14 | 0.49 | 0.15 | 0.10 | 0.19 | 0.40 | 0.08 | 0.39 | 0.59 |
| 22 | 0.31 | | 0.18 | 0.46 | 0.13 | 0.09 | 0.22 | 0.29 | 0.12 | 0.33 | 0.50 |
| 23 | 0.31 | | 0.17 | 0.45 | 0.11 | 0.10 | 0.18 | 0.27 | 0.08 | 0.38 | 0.53 |
| 24 | 0.40 | | 0.12 | 0.46 | 0.18 | 0.09 | 0.24 | 0.39 | 0.08 | 0.32 | 0.52 |
| 25 | 0.50 | | 0.14 | 0.55 | 0.14 | 0.11 | 0.21 | 0.36 | 0.08 | 0.45 | 0.67 |
| 26 | 0.30 | | 0.15 | 0.54 | 0.18 | 0.10 | 0.23 | 0.30 | 0.08 | 0.35 | 0.49 |
| 27 | 0.34 | | 0.26 | 0.59 | 0.40 | 0.10 | 0.65 | 0.31 | 0.08 | 0.42 | 0.55 |
| 28 | 1.29 | | 0.23 | 0.65 | 0.24 | 0.31 | 0.46 | 1.40 | 0.10 | 0.64 | 0.74 |
| 29 | 1.21 | | 0.16 | 0.52 | 0.41 | 0.21 | 0.74 | 1.34 | 0.10 | 0.55 | 0.64 |
| 30 | 1.13 | | 0.23 | 0.70 | 0.16 | 0.27 | 0.26 | 1.25 | 0.10 | 0.59 | 0.40 |
| 31 | 0.60 | | 0.14 | 0.52 | 0.19 | 0.10 | 0.30 | 0.50 | 0.09 | 0.61 | 0.27 |
| 32 | 0.51 | | 0.18 | 0.57 | 0.23 | 0.12 | 0.21 | 0.56 | 0.12 | 0.41 | 0.72 |
| 33 | 0.38 | | 0.18 | 0.51 | 0.20 | 0.10 | 0.61 | 0.45 | 0.11 | 0.31 | 0.61 |
| 34 | 1.00 | | 0.22 | 0.48 | 0.14 | 0.14 | 0.39 | 0.92 | 0.17 | 0.43 | 0.70 |
| 35 | 0.71 | | 0.13 | 0.51 | 0.12 | 0.10 | 0.22 | 0.56 | 0.08 | 0.42 | 0.59 |
| 36 | 0.48 | | 0.25 | 0.51 | 0.22 | 0.09 | 0.16 | 0.49 | 0.09 | 0.37 | 0.67 |
| 37 | 0.48 | | 0.15 | 0.62 | 0.10 | 0.14 | 0.12 | 0.45 | 0.14 | 0.43 | 0.60 |
| 38 | 0.26 | | 0.13 | 0.53 | 0.13 | 0.08 | 0.12 | 0.24 | 0.07 | 0.31 | 0.50 |
| 39 | 0.25 | | 0.10 | 0.48 | 0.17 | 0.07 | 0.23 | 0.22 | 0.07 | 0.30 | 0.52 |
| 40 | 0.44 | | 0.18 | 0.56 | 0.18 | 0.09 | 0.16 | 0.41 | 0.08 | 0.36 | 0.40 |
| 41 | 0.30 | | 0.11 | 0.54 | 0.26 | 0.08 | 0.14 | 0.23 | 0.16 | 0.33 | 0.43 |
| 42 | 0.51 | | 0.16 | 0.58 | 0.19 | 0.12 | 0.21 | 0.47 | 0.11 | 0.38 | 0.71 |
| 43 | 0.44 | | 0.15 | 0.54 | 0.13 | 0.10 | 0.19 | 0.32 | 0.08 | 0.43 | 0.36 |
| 44 | 0.50 | | 0.14 | 0.57 | 0.19 | 0.09 | 0.20 | 0.44 | 0.09 | 0.50 | 0.59 |
| 45 | 0.46 | | 0.17 | 0.58 | 0.32 | 0.09 | 0.57 | 0.47 | 0.13 | 0.40 | 0.66 |
| 46 | 1.20 | | 0.28 | 0.55 | 0.27 | 0.25 | 0.47 | 1.33 | 0.23 | 0.51 | 0.74 |
| 47 | 1.17 | | 0.15 | 0.53 | 0.16 | 0.24 | 0.31 | 1.28 | 0.12 | 0.56 | 0.78 |
| 48 | 1.02 | | 0.19 | 0.53 | 0.30 | 0.15 | 0.12 | 0.96 | 0.08 | 0.49 | 0.60 |
| 49 | 0.77 | | 0.16 | 0.63 | 0.10 | 0.10 | 0.10 | 0.63 | 0.15 | 0.44 | 0.67 |
| 50 | 0.39 | | 0.11 | 0.63 | 0.31 | 0.08 | 0.22 | 0.37 | 0.09 | 0.35 | 0.47 |
| 51 | 0.38 | | 0.15 | 0.48 | 0.25 | 0.06 | 0.16 | 0.31 | 0.06 | 0.38 | 0.58 |
| 52 | 0.34 | | 0.20 | 0.66 | 0.20 | 0.04 | 0.15 | 0.29 | 0.07 | 0.37 | 0.66 |
| 53 | 0.30 | | 0.16 | 0.54 | 0.27 | 0.11 | 0.23 | 0.24 | 0.13 | 0.40 | 0.50 |
| 54 | 0.27 | | 0.15 | 0.09 | 0.17 | 0.18 | 0.11 | 0.22 | 0.08 | 0.32 | 0.58 |
| 55 | 0.22 | | 0.22 | 0.53 | 0.15 | 0.28 | 0.24 | 0.19 | 0.04 | 0.33 | 0.65 |
| 56 | 0.34 | | 0.17 | 0.64 | 0.22 | 0.09 | 0.37 | 0.32 | 0.12 | 0.35 | 0.57 |
| 57 | 0.39 | | 0.14 | 0.58 | 0.10 | 0.05 | 0.12 | 0.35 | 0.11 | 0.41 | 0.62 |
| 58 | 0.41 | | 0.16 | 0.50 | 0.18 | 0.22 | 0.15 | 0.24 | 0.07 | 0.35 | 0.45 |
| 59 | 0.30 | | 0.15 | 0.47 | 0.13 | 0.18 | 0.17 | 0.29 | 0.09 | 0.40 | 0.62 |
| 60 | 0.31 | | 0.20 | 0.40 | 0.11 | 0.09 | 0.11 | 0.33 | 0.13 | 0.34 | 0.70 |
| **BLANK** | | 0.003 | 0.012 | 0.007 | 0.011 | 0.009 | 0.006 | 0.019 | 0.014 | 0.024 | 0.003 |

*Blank is the signal obtained using incubation buffer instead of serum.

**Table D. IgM** **ELISA results for LS.**

| %/A_450_ | **Antigen:**  D1D | | L1D | D2D | L2D | D3D | L3D | R1 | R1D | C1 | C2 |
| --- | --- | --- | --- | --- | --- | --- | --- | --- | --- | --- | --- |
| 1 | 0.79 | | 0.38 | 0.53 | 0.77 | 0.25 | 0.75 | 0.89 | 0.25 | 0.34 | 0.47 |
| 2 | 0.87 | | 0.48 | 0.65 | 0.66 | 0.39 | 0.92 | 0.89 | 0.51 | 0.38 | 0.67 |
| 3 | 0.37 | | 0.16 | 0.28 | 0.32 | 0.11 | 0.26 | 0.39 | 0.10 | 0.15 | 0.17 |
| 4 | 0.36 | | 0.15 | 0.23 | 0.23 | 0.10 | 0.33 | 0.41 | 0.10 | 0.13 | 0.14 |
| 5 | 1.05 | | 0.45 | 0.65 | 0.65 | 0.26 | 0.60 | 1.01 | 0.17 | 0.49 | 0.30 |
| 6 | 0.99 | | 0.32 | 0.51 | 0.40 | 0.18 | 0.56 | 0.76 | 0.14 | 0.37 | 0.22 |
| 7 | 0.87 | | 0.29 | 0.40 | 0.40 | 0.09 | 0.51 | 0.97 | 0.10 | 0.37 | 0.21 |
| 8 | 0.52 | | 0.34 | 0.40 | 0.32 | 0.15 | 0.37 | 0.64 | 0.07 | 0.19 | 0.14 |
| 9 | 0.46 | | 0.28 | 0.30 | 0.45 | 0.12 | 0.37 | 0.53 | 0.22 | 0.16 | 0.16 |
| 10 | 0.87 | | 0.30 | 0.34 | 0.69 | 0.16 | 0.50 | 0.92 | 0.18 | 0.30 | 0.23 |
| 11 | 0.51 | | 0.21 | 0.76 | 0.39 | 0.11 | 0.36 | 0.60 | 0.10 | 0.18 | 0.17 |
| 12 | 0.32 | | 0.20 | 0.18 | 0.36 | 0.09 | 0.32 | 0.37 | 0.11 | 0.13 | 0.15 |
| 13 | 0.26 | | 0.15 | 0.16 | 0.19 | 0.09 | 0.23 | 0.32 | 0.12 | 0.09 | 0.14 |
| 14 | 0.89 | | 0.40 | 0.66 | 0.67 | 0.22 | 0.80 | 0.88 | 0.20 | 0.32 | 0.37 |
| 15 | 0.91 | | 0.38 | 0.67 | 0.65 | 0.24 | 0.73 | 0.86 | 0.22 | 0.34 | 0.39 |
| 16 | 0.57 | | 0.21 | 0.45 | 0.60 | 0.16 | 0.64 | 0.65 | 0.15 | 0.24 | 0.23 |
| 17 | 0.64 | | 0.37 | 0.47 | 0.47 | 0.18 | 0.69 | 0.61 | 0.14 | 0.28 | 0.23 |
| 18 | 0.96 | | 0.28 | 0.47 | 0.45 | 0.44 | 0.62 | 0.67 | 0.19 | 0.71 | 0.24 |
| 19 | 0.77 | | 0.19 | 0.39 | 0.42 | 0.22 | 0.46 | 0.85 | 0.14 | 0.41 | 0.22 |
| 20 | 0.83 | | 0.48 | 0.52 | 0.35 | 0.25 | 0.53 | 0.87 | 0.17 | 0.41 | 0.24 |
| 21 | 0.87 | | 0.36 | 0.41 | 0.70 | 0.22 | 0.57 | 0.90 | 0.15 | 0.44 | 0.27 |
| 22 | 0.58 | | 0.27 | 0.37 | 0.49 | 0.18 | 0.54 | 0.67 | 0.07 | 0.29 | 0.22 |
| 23 | 0.58 | | 0.33 | 1.58 | 0.60 | 0.18 | 0.56 | 0.58 | 0.26 | 0.26 | 0.28 |
| 24 | 0.95 | | 0.37 | 0.34 | 0.74 | 0.22 | 0.61 | 0.87 | 0.21 | 0.45 | 0.41 |
| 25 | 0.49 | | 0.28 | 0.45 | 0.51 | 0.16 | 0.60 | 0.53 | 0.11 | 0.19 | 0.20 |
| 26 | 0.57 | | 0.20 | 0.37 | 0.43 | 0.14 | 0.43 | 0.58 | 0.11 | 0.19 | 0.25 |
| 27 | 0.52 | | 0.18 | 0.34 | 0.38 | 0.12 | 0.41 | 0.57 | 0.18 | 0.17 | 0.19 |
| 28 | 1.50 | | 0.48 | 0.89 | 1.00 | 0.95 | 1.30 | 1.48 | 0.39 | 0.90 | 0.63 |
| 29 | 1.23 | | 0.26 | 0.50 | 0.44 | 0.65 | 0.99 | 1.01 | 0.14 | 0.69 | 0.31 |
| 30 | 1.06 | | 0.62 | 1.00 | 0.80 | 1.08 | 1.42 | 1.19 | 0.30 | 0.93 | 0.64 |
| 31 | 0.97 | | 0.34 | 0.52 | 0.64 | 0.28 | 0.70 | 1.05 | 0.16 | 0.41 | 0.28 |
| 32 | 0.92 | | 0.20 | 0.57 | 0.38 | 0.23 | 0.44 | 1.06 | 0.25 | 0.25 | 0.24 |
| 33 | 0.48 | | 0.25 | 0.29 | 0.42 | 0.13 | 0.41 | 0.57 | 0.16 | 0.14 | 0.20 |
| 34 | 0.88 | | 0.25 | 0.38 | 0.46 | 0.37 | 1.02 | 0.99 | 0.15 | 0.67 | 0.26 |
| 35 | 0.62 | | 0.24 | 0.33 | 0.44 | 0.19 | 0.61 | 0.66 | 0.13 | 0.36 | 0.21 |
| 36 | 0.65 | | 0.38 | 0.36 | 0.70 | 0.15 | 0.53 | 0.76 | 0.24 | 0.19 | 0.21 |
| 37 | 1.08 | | 0.79 | 0.78 | 0.91 | 0.46 | 0.95 | 0.93 | 0.53 | 0.60 | 0.63 |
| 38 | 0.56 | | 0.22 | 0.42 | 0.53 | 0.14 | 0.41 | 0.58 | 0.12 | 0.18 | 0.22 |
| 39 | 0.76 | | 0.23 | 0.32 | 0.41 | 0.13 | 0.40 | 0.68 | 0.13 | 022 | 0.12 |
| 40 | 0.63 | | 0.23 | 0.36 | 0.35 | 0.14 | 0.44 | 0.61 | 0.12 | 0.20 | 0.18 |
| 41 | 0.45 | | 0.18 | 0.30 | 0.35 | 0.12 | 0.37 | 0.47 | 0.10 | 0.14 | 0.17 |
| 42 | 0.63 | | 0.37 | 0.36 | 0.59 | 0.17 | 0.44 | 0.65 | 0.14 | 0.12 | 0.22 |
| 43 | 0.86 | | 0.37 | 0.45 | 0.69 | 0.19 | 0.62 | 0.74 | 0.15 | 0.19 | 0.30 |
| 44 | 0.91 | | 0.26 | 0.29 | 0.30 | 0.14 | 0.28 | 0.80 | 0.11 | 0.21 | 0.15 |
| 45 | 0.98 | | 0.24 | 0.34 | 0.45 | 0.14 | 0.52 | 0.91 | 0.18 | 0.22 | 0.25 |
| 46 | 1.76 | | 0.47 | 0.65 | 0.93 | 0.66 | 1.01 | 1.43 | 0.27 | 0.68 | 0.46 |
| 47 | 1.77 | | 0.39 | 0.51 | 0.88 | 0.49 | 0.94 | 1.37 | 0.24 | 0.67 | 0.42 |
| 48 | 1.57 | | 0.23 | 0.27 | 0.49 | 0.28 | 0.69 | 1.18 | 0.11 | 0.54 | 0.24 |
| 49 | 0.81 | | 0.22 | 0.30 | 0.34 | 0.26 | 0.45 | 0.95 | 0.10 | 0.39 | 0.16 |
| 50 | 0.42 | | 0.20 | 0.38 | 0.38 | 0.11 | 0.35 | 0.51 | 0.13 | 0.11 | 0.18 |
| 51 | 0.44 | | 0.20 | 0.32 | 0.42 | 0.11 | 0.36 | 0.46 | 0.10 | 0.21 | 0.19 |
| 52 | 0.39 | | 0.21 | 0.32 | 0.34 | 0.12 | 0.37 | 0.43 | 0.14 | 0.22 | 0.21 |
| 53 | 0.41 | | 0.23 | 0.30 | 0.39 | 0.10 | 0.15 | 0.42 | 0.17 | 0.13 | 0.18 |
| 54 | 0.33 | | 0.16 | 0.19 | 0.24 | 0.08 | 0.20 | 0.39 | 0.11 | 0.08 | 0.11 |
| 55 | 0.30 | | 0.16 | 0.19 | 0.27 | 0.09 | 0.23 | 0.40 | 0.10 | 0.11 | 0.13 |
| 56 | 0.39 | | 0.32 | 0.37 | 0.52 | 0.16 | 0.27 | 0.64 | 0.16 | 0.21 | 0.28 |
| 57 | 0.71 | | 0.28 | 0.40 | 0.49 | 0.20 | 0.54 | 0.76 | 0.14 | 0.17 | 0.26 |
| 58 | 0.23 | | 0.11 | 0.15 | 0.20 | 0.16 | 0.18 | 0.27 | 0.08 | 0.15 | 0.11 |
| 59 | 0.25 | | 0.14 | 0.15 | 0.26 | 0.08 | 0.21 | 0.32 | 0.10 | 0.14 | 0.09 |
| 60 | 0.46 | | 0.39 | 0.31 | 0.49 | 0.13 | 0.51 | 0.48 | 0.24 | 0.16 | 0.23 |
| **BLANK** | | 0.014 | 0.021 | 0.016 | 0.006 | 0.008 | 0.014 | 0.022 | 0.024 | 0.015 | 0.013 |

*Blank is the signal obtained using incubation buffer instead of serum.
