# Supplementary material for: Detection of autoimmune antibodies in localized scleroderma by synthetic oligonucleotide antigens
Source: PLoS One. 2018 Apr 11;13(4):e0195381. doi: 10.1371/journal.pone.0195381 (PMC5895021; doi:10.1371/journal.pone.0195381)
Supplement: S3 Appendix — (DOCX) [file pone.0195381.s003.docx]

# List of abbreviations

**General**

LS – localized scleroderma

pSLE – pediatric systemic lupus erythematosus

polyJIA – polyarticular juvenile idiopathic arthritis

SLEDAI – systemic lupus erythematosus disease activity index

ANA – antinuclear antibody

IgG – immunoglobulin class G

IgM – immunoglobulin class M

ELISA – enzyme-linked immunosorbent assay

HPR – horseradish peroxidase

CTD – calf thymus DNA

nt – nucleotide

LNA – locked nucleic acid

| mLOSSI - modified Localized Scleroderma Skin Score |
| --- |
| LOSDI - Localized Scleroderma Damage Index |
| PGAACT - Physician Global Assesment Disease |
| PGADAMG - Physician Global Assesment Disease |

# Demographic and clinical characteristics of subjects used in this study

**Table A.** **Demographic and clinical characteristics of LS and pSLE subjects.**

| **Variable** | **LS (n=30, 60 samples)** | **pSLE subjects (n=27, 30 samples)** |
| --- | --- | --- |
| Female/male | 21/9 | 24/3 |
| Hispanic (%) | 3 (11) | 16 (59) |
| Asian/Pacific Islander (%) | 0 (0) | 5 (19) |
| Non-Hispanic Caucasian (%) | 29 (96.7) | 6 (22) |
| African-American (%) | 1 (3.3) | 0 (0) |
| Age at diagnosis, median (range) years | 9.80 (4.00-18.09) | 14.1 (7.5-17.6) |
| Age at sample collection, median, (range) years | 13.22 (5.59-21.71) | 14.5 (7.5-19.2) |
| ss/dsDNA antibody positive patients at onset – ELISA for LS, *Crithidia assay* for pSLE (%) | 7 (23%) | 12 (40) |
| ANA positive at sampling (% all samples) | 3 (5)* | 26 (87) |
| Anti-histone positive at sampling(%) | 12 (20) | nd |
| Active disease, no. samples (%) | 30 (50) | 30 (100) |
| Class III/IV lupus nephritis-biopsy proven† (%) | na | 10 (33) |
| Mean (range) SLEDAI score | na | 11.6 (4-30) |
| Mean (range) ESR | na | 48 (1-132) |
| Mean (range) C3 complement | na | 72 (24-121) |
| mLOSSI median (range) | 3 (0-45) | na |
| LOSDI median (range) | 5.5 (1-61) | na |
| PGAACT median (range) | 1.5 (0-100) | na |
| PGADAMG median (range) | 30 (0-60) | na |
| Time in treatment, years, median (range) | 1.8 (0.01-13.75) | 0.13 (0-17.3) |
| Methotrexate at sample (%) | 38 (63%) | 2 (6.7) |
| Other medication at sample (%) | 17 (28%) | 29 (97) |

For symbols, see Abbreviations. Na = not applied; nd = no data. Other LS medication: Prednisone/prednisolone, topical corticosteroid, cellcept, doxycycline, solumedrol, planquenil, myfortic. SLE other medication: prednisone, HCQ, IV solumedrol, cellcept, ranitidine, piroxicam.

* Clinical ANA data for LS patients: 5 speckled, 2 homogenous, 1 nucleolar; ANA levels: 1:80 (n=2; speckled and nucleolar), 1:160 (n=3, 2 speckled and 1 homogenous), 1:320 (n=2, speckled and homogenous), 1:640 (n=1, homogenous).

| **Table B. Disease subtype and medication usage for LS patients.** | |
| --- | --- |
| Disease Subtype, n (%) | LS patients (n=30)^1^ |
| Linear trunk/limb (t/l) | 10 (33) |
| Linear face/scalp (f/s) | 6 (20) |
| Deep morphea | 1( 3.3) |
| Plaque morphea | 4 (13) |
| Generalized morphea | 8 (27) |
| Eosinophilic fasciitis | 1 (3.3) |
| On systemic treatment at **any** visit, n (%)^2^ | 28 (93) |
| On systemic treatment at active visit | 10 (33) |
| On systemic treatment at inactive visit | 28 (93) |
| ^1^ Each patient had 2 samples for a total of 60 samples.  ^2^Of patients on therapy, the majority have used methotrexate (MTX) (71%), followed by a few subjects on prednisone (n=10) and cellcept (n=6). (Some patients have been on combination therapy). |  |


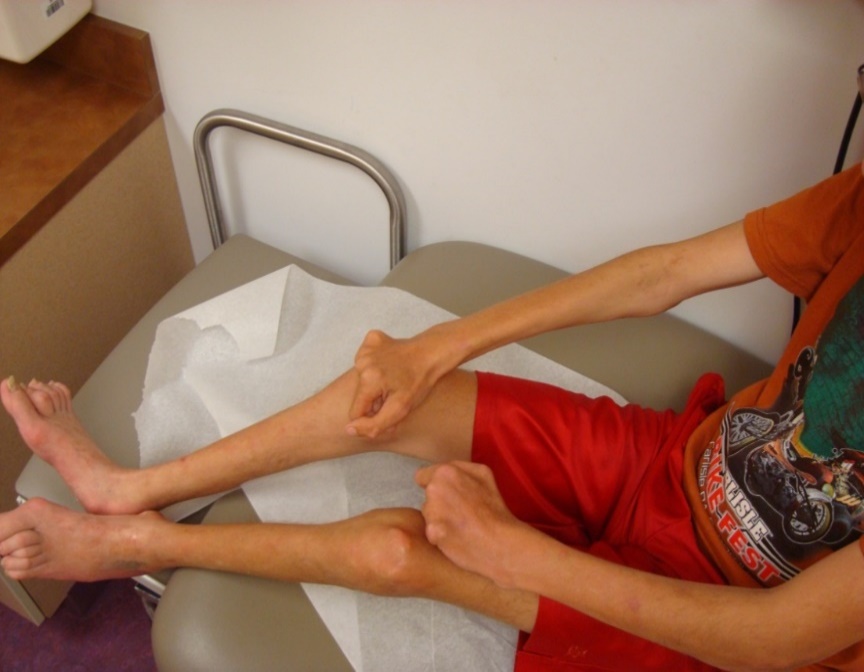


A

B


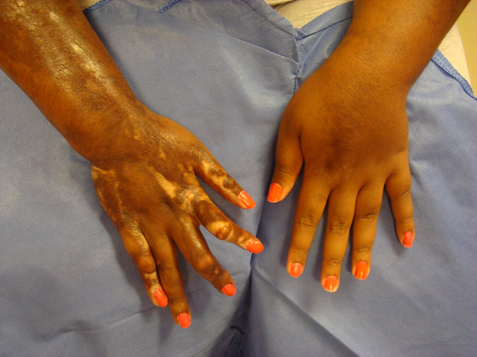


C


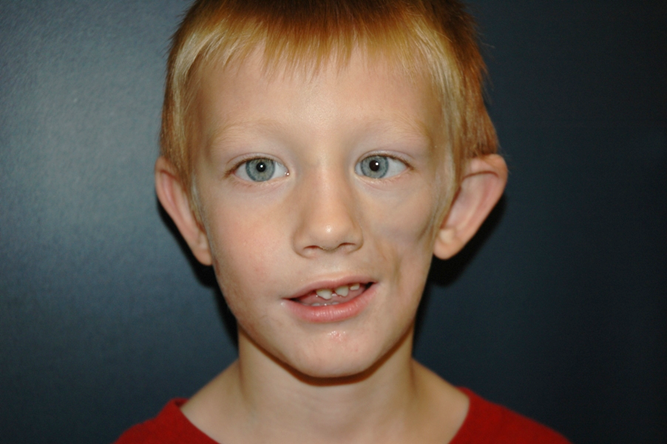


D

**Figure A. Pediatric localized scleroderma subtypes.** A) Pansclerotic morphea affecting limbs circumferentially and sparing distal toes and fingers. B) En coup de sabre (ECDS) lesion affecting the scalp and head in a unilateral fashion. C) Linear scleroderma lesion affecting unilateral right upper extremity with associated joint contractures of the wrist, metacarpal and phalangeal joints. D) Parry Romberg Syndrome/ hemifacial atrophy of the left side of the face.

**Table C.** **Demographic and clinical characteristics of healthy control subjects.**

| **Variable** | **Healthy control (HC), adults** | **Healthy control (HC), children** |
| --- | --- | --- |
| N (subjects) | 60 | 15 |
| Female/Male | 44/16 | 10/5 |
| African-American | 0 | 1 |
| Asian/Pacific islander | 2 | 0 |
| Caucasian | 58 | 14 |
| Caucasian Hispanic | 0 | 0 |
| Ethnicity unknown | 0 | 0 |
| Median age (yr) at sample collection (range) | 42 (17.0-53.0) | 10 (8.6-14.0) |

na = not applicable

# Results of ELISA for pSLE cohort


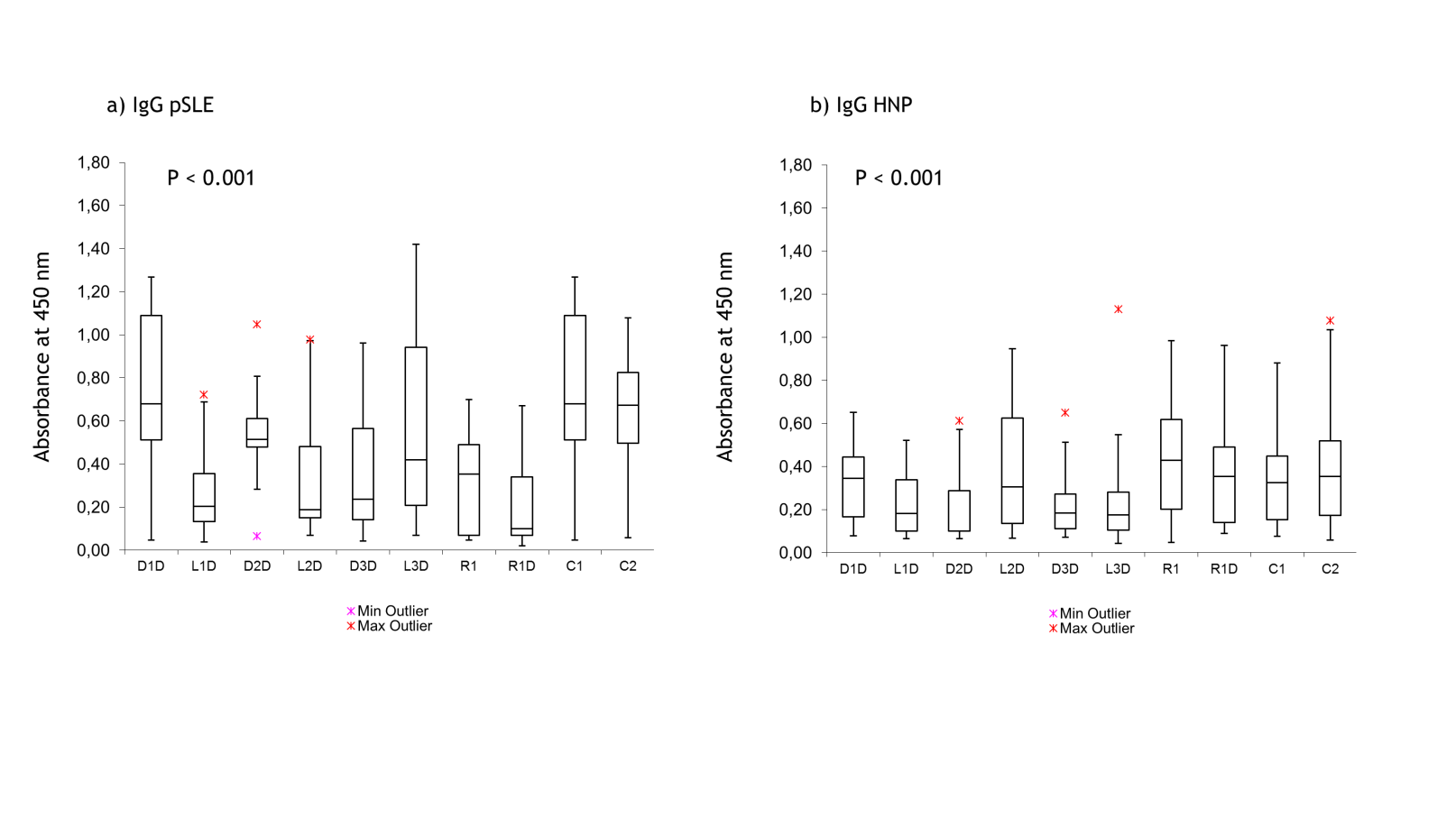


**Figure B. Results of ELISA assay.** The results for pediatric SLE subjects (n = 27) are shown as a box-and-whisker plot with outliers (indicated as stars). The arms on each boxplot are values Q1 – 1.5 × IQR and Q3 + 1.5 × IQR. Data points for each subject are means for three independent measurements.

# Sensitivity assessment for D1D and commercial a-ssDNA ELISA

a)

b)

**Figure C. Sensitivity of commercial a-ssDNA vs. D1D ELISA.^[[1]](#footnote-1)^**

1. Bio-Rad kit (#96SS) was used for anti-ssDNA test. For the assay, we used 5 randomly patient samples. [↑](#footnote-ref-1)
