# Supplementary material for: Detection of autoimmune antibodies in localized scleroderma by synthetic oligonucleotide antigens
Source: PLoS One. 2018 Apr 11;13(4):e0195381. doi: 10.1371/journal.pone.0195381 (PMC5895021; doi:10.1371/journal.pone.0195381)
Supplement: S4 Appendix — (DOCX) [file pone.0195381.s004.docx]

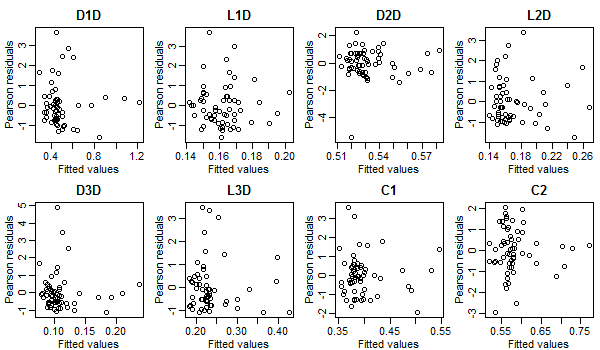


**Figure A. Pearson residuals versus fitted values for IgG LS.**

**Response variable D1D**

|  | ***Estimate*** | ***Std. error*** | ***Z-value*** | ***p*-value** |
| --- | --- | --- | --- | --- |
| (Intercept) | -0.7371 | 0.1481 | -4.9783 | < 0.001 |
| Time in Treatment | -0.0424 | 0.0175 | -2.4176 | 0.0156 |
| mLOSSI | -0.0076 | 0.0077 | -0.9843 | 0.3250 |
| LOSDI | 0.0205 | 0.0042 | 4.8415 | < 0.001 |
| PGAACT | -0.0009 | 0.0024 | -0.3514 | 0.7253 |
| PGADAMG | -0.0024 | 0.0043 | -0.5631 | 0.5734 |

**Table A: Regression coefficients for D1D, IgG LS data.**

**Response variable L1D**

|  | ***Estimate*** | ***Std. error*** | ***Z-value*** | ***p*-value** |
| --- | --- | --- | --- | --- |
| (Intercept) | 0.1646 | 0.0142 | 11.6298 | < 0.001 |
| Time in Treatment | -0.0007 | 0.0016 | -0.4165 | 0.6771 |
| mLOSSI | 0.0003 | 0.0008 | 0.4081 | 0.6832 |
| LOSDI | 0.0006 | 0.0005 | 1.2079 | 0.2271 |
| PGAACT | -0.0003 | 0.0002 | -1.2620 | 0.2070 |
| PGADAMG | -0.0001 | 0.0004 | -0.2564 | 0.7976 |

**Table B: Regression coefficients for L1D, IgG LS data.**

**Response variable D2D**

|  | ***Estimate*** | ***Std. error*** | ***Z-value*** | ***p*-value** |
| --- | --- | --- | --- | --- |
| (Intercept) | 0.5200 | 0.0299 | 17.3913 | < 0.001 |
| Time in Treatment | -0.0014 | 0.0034 | -0.4142 | 0.6787 |
| mLOSSI | -0.0013 | 0.0017 | -0.7758 | 0.4379 |
| LOSDI | 0.0015 | 0.0010 | 1.4855 | 0.1374 |
| PGAACT | -0.0001 | 0.0005 | -0.2991 | 0.7648 |
| PGADAMG | 0.0003 | 0.0009 | 0.2999 | 0.7642 |

**Table C: Regression coefficients for D2D, IgG LS data.**

**Response variable L2D**

|  | ***Estimate*** | ***Std. error*** | ***Z-value*** | ***p*-value** |
| --- | --- | --- | --- | --- |
| (Intercept) | -1.8976 | 0.1344 | -14.1204 | < 0.001 |
| Time in Treatment | 0.0008 | 0.0151 | 0.0561 | 0.9553 |
| mLOSSI | -0.0110 | 0.0075 | -1.4643 | 0.1431 |
| LOSDI | 0.0138 | 0.0043 | 3.2104 | 0.0013 |
| PGAACT | -0.0011 | 0.0022 | -0.5059 | 0.6129 |
| PGADAMG | 0.0007 | 0.0039 | 0.1776 | 0.8591 |

**Table D: Regression coefficients for L2D, IgG LS data.**

**Response variable D3D**

|  | ***Estimate*** | ***Std. error*** | ***Z-value*** | ***p*-value** |
| --- | --- | --- | --- | --- |
| (Intercept) | -2.1877 | 0.1208 | -18.1131 | < 0.001 |
| Time in Treatment | -0.0258 | 0.0123 | -2.1080 | 0.0350 |
| mLOSSI | 0.0045 | 0.0080 | 0.5624 | 0.5739 |
| LOSDI | 0.0135 | 0.0046 | 2.9680 | 0.0030 |
| PGAACT | -0.0003 | 0.0019 | -0.1473 | 0.8829 |
| PGADAMG | -0.0051 | 0.0033 | -1.5495 | 0.1213 |

**Table E: Regression coefficients for D3D, IgG LS data.**

**Response variable L3D**

|  | ***Estimate*** | ***Std. error*** | ***Z-value*** | ***p*-value** |
| --- | --- | --- | --- | --- |
| (Intercept) | -1.5017 | 0.1847 | -8.1320 | < 0.001 |
| Time in Treatment | -0.0241 | 0.0201 | -1.2010 | 0.2297 |
| mLOSSI | -0.0147 | 0.0100 | -1.4727 | 0.1408 |
| LOSDI | 0.0165 | 0.0070 | 2.3743 | 0.0176 |
| PGAACT | 0.0011 | 0.0031 | 0.3488 | 0.7273 |
| PGADAMG | -0.0010 | 0.0053 | -0.1842 | 0.8538 |

**Table F: Regression coefficients for L3D, IgG LS data.**

**Response variable C1**

|  | ***Estimate*** | ***Std. error*** | ***Z-value*** | ***p*-value** |
| --- | --- | --- | --- | --- |
| (Intercept) | 0.3760 | 0.0250 | 15.0211 | < 0.001 |
| Time in Treatment | -0.0034 | 0.0028 | -1.2106 | 0.2260 |
| mLOSSI | 0.0004 | 0.0014 | 0.3012 | 0.7632 |
| LOSDI | 0.0028 | 0.0008 | 3.5993 | < 0.001 |
| PGAACT | 0.0003 | 0.0004 | 0.6449 | 0.5190 |
| PGADAMG | -0.0003 | 0.0007 | -0.3967 | 0.6916 |

**Table G: Regression coefficients for C1, IgG LS data.**

**Response variable C2**

|  | ***Estimate*** | ***Std. error*** | ***Z-value*** | ***p*-value** |
| --- | --- | --- | --- | --- |
| (Intercept) | 0.5507 | 0.0336 | 16.4106 | < 0.001 |
| Time in Treatment | 0.0030 | 0.0038 | 0.7900 | 0.4295 |
| mLOSSI | 0.0043 | 0.0019 | 2.2929 | 0.0219 |
| LOSDI | 0.0015 | 0.0011 | 1.3533 | 0.1760 |
| PGAACT | 0.0006 | 0.0006 | 0.9920 | 0.3212 |
| PGADAMG | -0.0009 | 0.0010 | -0.8803 | 0.3787 |

**Table H: Regression coefficients for C2, IgG LS data.**


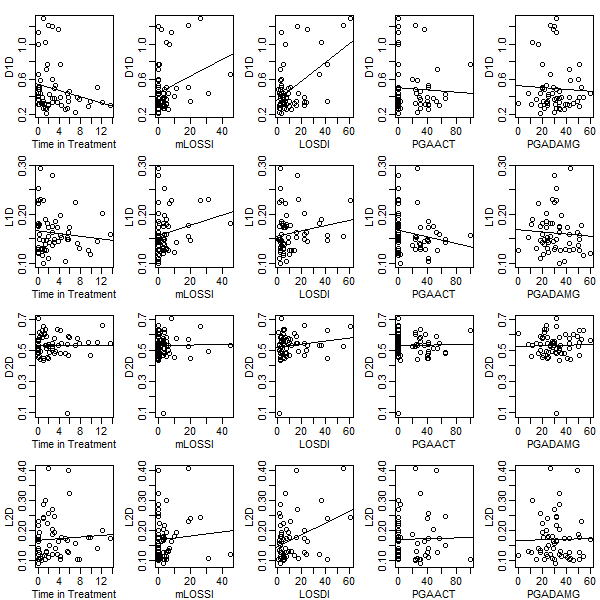


**Figure B. Scatterplots for pairwise comparison of the variables, IgG LS data.**


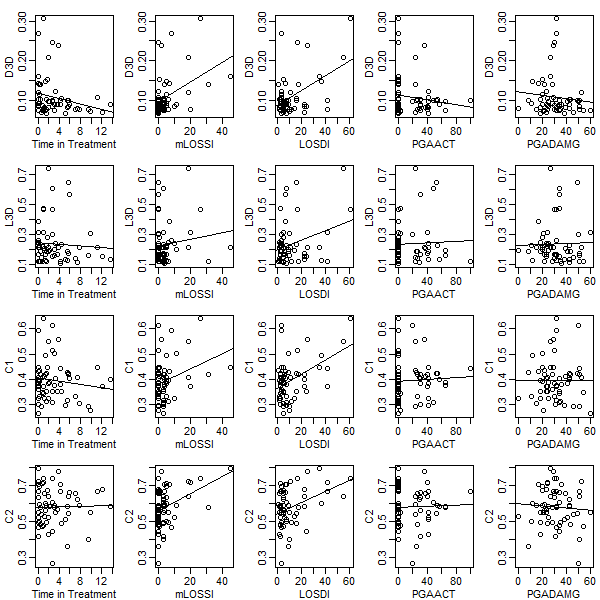


**Figure C. Scatterplots for pairwise comparison of the variables, IgG LS data (continued).**


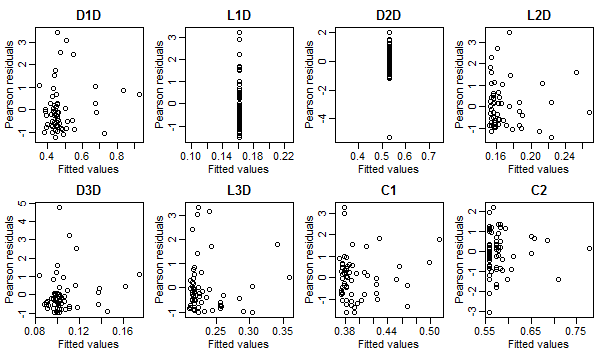


**Figure D. Pearson residuals versus fitted values, parsimonious IgG model.**

**.**

**Response variable D1D**

|  | ***Estimate*** | ***Std. error*** | ***Z-value*** | ***p*-value** |
| --- | --- | --- | --- | --- |
| (Intercept) | -0.8325 | 0.0749 | -11.1092 | < 0.001 |
| Time in Treatment | -0.0235 | 0.0101 | -2.3378 | 0.0194 |
| LOSDI | 0.0128 | 0.0041 | 3.1531 | 0.0016 |

**Table I: Regression coefficients for D1D, parsimonious IgG model.**

**Response variable L1D**

|  | ***Estimate*** | ***Std. error*** | ***Z-value*** | ***p*-value** |
| --- | --- | --- | --- | --- |
| (Intercept) | 0.1618 | 0.0052 | 30.8615 | < 0.001 |

**Table J Regression coefficients for L1D, parsimonious IgG model.**

**Response variable D2D**

|  | ***Estimate*** | ***Std. error*** | ***Z-value*** | ***p*-value** |
| --- | --- | --- | --- | --- |
| (Intercept) | 0.5318 | 0.0107 | 49.7488 | < 0.001 |

**Table K: Regression coefficients for D2D, parsimonious IgG model.**

**Response variable L2D**

|  | ***Estimate*** | ***Std. error*** | ***Z-value*** | ***p*-value** |
| --- | --- | --- | --- | --- |
| (Intercept) | -1.8958 | 0.0640 | -29.6040 | < 0.001 |
| LOSDI | 0.0095 | 0.0037 | 2.5847 | 0.0097 |

**Table L: Regression coefficients for L2D, parsimonious IgG model.**

**Response variable D3D**

|  | ***Estimate*** | ***Std. error*** | ***Z-value*** | ***p*-value** |
| --- | --- | --- | --- | --- |
| (Intercept) | -2.3181 | 0.0599 | -38.6987 | < 0.001 |
| Time in Treatment | -0.0179 | 0.0081 | -2.2016 | 0.0277 |
| LOSDI | 0.0096 | 0.0040 | 2.4293 | 0.0151 |

**Table M: Regression coefficients for D3D, parsimonious IgG model.**

**Response variable L3D**

|  | ***Estimate*** | ***Std. error*** | ***Z-value*** | ***p*-value** |
| --- | --- | --- | --- | --- |
| (Intercept) | -1.5658 | 0.0900 | -17.3994 | < 0.001 |
| LOSDI | 0.0089 | 0.0059 | 1.5111 | 0.1308 |

**Table N: Regression coefficients for L3D, parsimonious IgG model.**

**Response variable C1**

|  | ***Estimate*** | ***Std. error*** | ***Z-value*** | ***p*-value** |
| --- | --- | --- | --- | --- |
| (Intercept) | 0.3693 | 0.0116 | 31.8064 | < 0.001 |
| LOSDI | 0.0024 | 0.0006 | 3.8494 | < 0.001 |

**Table O: Regression coefficients for C1, parsimonious IgG model.**

**Response variable C2**

|  | ***Estimate*** | ***Std. error*** | ***Z-value*** | ***p*-value** |
| --- | --- | --- | --- | --- |
| (Intercept) | 0.5582 | 0.0139 | 40.2332 | < 0.001 |
| mLOSSI | 0.0049 | 0.0014 | 3.5310 | < 0.001 |

**Table P: Regression coefficients for C2, parsimonious IgG model.**

| **Covariate** | **VIF** |
| --- | --- |
| Time in Treatment | 1.2031 |
| mLOSSI | 1.8585 |
| LOSDI | 1.6306 |
| PGAACT | 1.2829 |
| PGADAMG | 1.1041 |

**Table Q: Variance Inflation Factors (VIF) for covariates.**


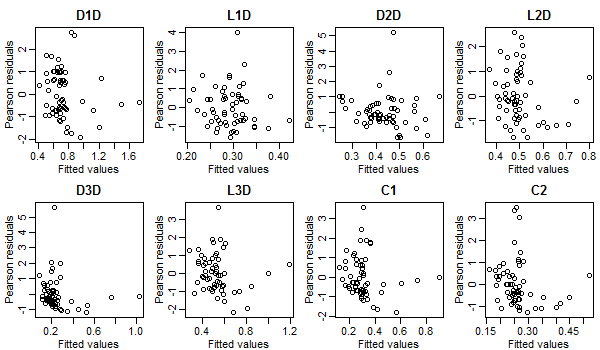


**Figure E. Pearson residuals versus fitted values, LS IgM data.**

**Response variable D1D**

|  | ***Estimate*** | ***Std. error*** | ***Z-value*** | ***p*-value** |
| --- | --- | --- | --- | --- |
| (Intercept) | -0.3795 | 0.1364 | -2.7819 | 0.0054 |
| Time in Treatment | -0.0474 | 0.0159 | -2.9858 | 0.0028 |
| mLOSSI | -0.0065 | 0.0077 | -0.8451 | 0.3981 |
| LOSDI | 0.0184 | 0.0042 | 4.3740 | < 0.001 |
| PGAACT | -0.0011 | 0.0022 | -0.5011 | 0.6163 |
| PGADAMG | 0.0006 | 0.0040 | 0.1469 | 0.8832 |

**Table R: Regression coefficients for D1D, LS IgM data.**

**Response variable L1D**

|  | ***Estimate*** | ***Std. error*** | ***Z-value*** | ***p*-value** |
| --- | --- | --- | --- | --- |
| (Intercept) | -1.1659 | 0.1293 | -9.0156 | < 0.001 |
| Time in Treatment | -0.0306 | 0.0138 | -2.2087 | 0.0272 |
| mLOSSI | 0.0059 | 0.0079 | 0.7433 | 0.4573 |
| LOSDI | 0.0015 | 0.0042 | 0.3451 | 0.7300 |
| PGAACT | -0.0008 | 0.0020 | -0.4015 | 0.6880 |
| PGADAMG | -0.0003 | 0.0038 | -0.0853 | 0.9320 |

**Table S: Regression coefficients for L1D, LS IgM data.**

**Response variable D2D**

|  | ***Estimate*** | ***Std. error*** | ***Z-value*** | ***p*-value** |
| --- | --- | --- | --- | --- |
| (Intercept) | 0.3261 | 0.0763 | 4.2742 | < 0.001 |
| Time in Treatment | -0.0188 | 0.0087 | -2.1757 | 0.0296 |
| mLOSSI | 0.0003 | 0.0043 | 0.0666 | 0.9469 |
| LOSDI | 0.0039 | 0.0025 | 1.5820 | 0.1136 |
| PGAACT | 0.0002 | 0.0012 | 0.1757 | 0.8605 |
| PGADAMG | 0.0036 | 0.0022 | 1.6458 | 0.0998 |

**Table T: Regression coefficients for D2D, LS IgM data.**

**Response variable L2D**

|  | ***Estimate*** | ***Std. error*** | ***Z-value*** | ***p*-value** |
| --- | --- | --- | --- | --- |
| (Intercept) | -0.8130 | 0.1211 | -6.7120 | < 0.001 |
| Time in Treatment | -0.0241 | 0.0140 | -1.7196 | 0.0855 |
| mLOSSI | 0.0048 | 0.0066 | 0.7279 | 0.4666 |
| LOSDI | 0.0066 | 0.0037 | 1.7820 | 0.0748 |
| PGAACT | -0.0005 | 0.0019 | -0.2387 | 0.8114 |
| PGADAMG | 0.0027 | 0.0035 | 0.7688 | 0.4420 |

**Table U: Regression coefficients for L2D, LS IgM data.**

**Response variable D3D**

|  | ***Estimate*** | ***Std. error*** | ***Z-value*** | ***p*-value** |
| --- | --- | --- | --- | --- |
| (Intercept) | -1.5817 | 0.2202 | -7.1829 | < 0.001 |
| Time in Treatment | -0.0808 | 0.0258 | -3.1384 | 0.0017 |
| mLOSSI | -0.0059 | 0.0125 | -0.4677 | 0.6400 |
| LOSDI | 0.0297 | 0.0067 | 4.4199 | < 0.001 |
| PGAACT | -0.0033 | 0.0035 | -0.9477 | 0.3433 |
| PGADAMG | 0.0008 | 0.0064 | 0.1278 | 0.8983 |

**Table V: Regression coefficients for D3D, LS IgM data.**

**Response variable L3D**

|  | ***Estimate*** | ***Std. error*** | ***Z-value*** | ***p*-value** |
| --- | --- | --- | --- | --- |
| (Intercept) | -0.6785 | 0.1451 | -4.6761 | < 0.001 |
| Time in Treatment | -0.0523 | 0.0225 | -2.3292 | 0.0198 |
| mLOSSI | -0.0051 | 0.0068 | -0.7478 | 0.4546 |
| LOSDI | 0.0160 | 0.0032 | 4.9349 | < 0.001 |
| PGAACT | -0.0023 | 0.0022 | -1.0061 | 0.3144 |
| PGADAMG | 0.0017 | 0.0042 | 0.4033 | 0.6867 |

**Table W: Regression coefficients for L3D, LS IgM data.**

**Response variable C1**

|  | ***Estimate*** | ***Std. error*** | ***Z-value*** | ***p*-value** |
| --- | --- | --- | --- | --- |
| (Intercept) | -1.1933 | 0.1820 | -6.5568 | < 0.001 |
| Time in Treatment | -0.0820 | 0.0284 | -2.8869 | 0.0039 |
| mLOSSI | -0.0091 | 0.0091 | -1.0055 | 0.3147 |
| LOSDI | 0.0231 | 0.0043 | 5.3164 | < 0.001 |
| PGAACT | -0.0019 | 0.0029 | -0.6798 | 0.4966 |
| PGADAMG | -0.0001 | 0.0054 | -0.0250 | 0.9801 |

**Table X: Regression coefficients for C1, LS IgM data.**

**Response variable C2**

|  | ***Estimate*** | ***Std. error*** | ***Z-value*** | ***p*-value** |
| --- | --- | --- | --- | --- |
| (Intercept) | -1.4333 | 0.1519 | -9.4355 | < 0.001 |
| Time in Treatment | -0.0427 | 0.0165 | -2.5944 | 0.0095 |
| mLOSSI | 0.0024 | 0.0095 | 0.2576 | 0.7967 |
| LOSDI | 0.0115 | 0.0052 | 2.2000 | 0.0278 |
| PGAACT | -0.0002 | 0.0024 | -0.0808 | 0.9356 |
| PGADAMG | 0.0018 | 0.0044 | 0.4040 | 0.6862 |

**Table Y: Regression coefficients for C2, LS IgM data.**


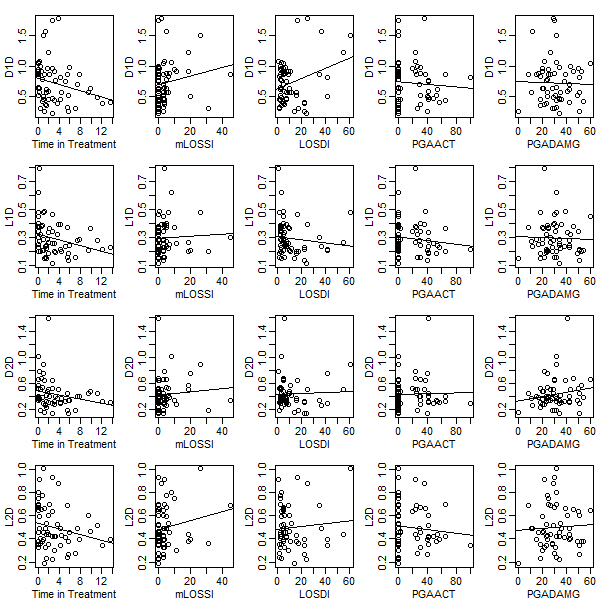


**Figure F. Scatterplots for pairwise comparison of the variables, LS IgM data.**


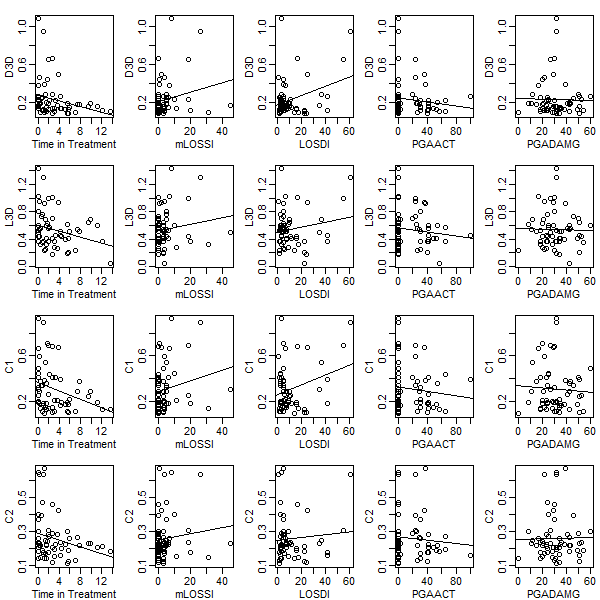


**Figure G. Scatterplots for pairwise comparison of the variables, LS IgM data (continued).**
